# The Relationships between physical activity, sedentary behaviour, sleep, and dementia: A systematic review and meta-analysis of cohort studies

**DOI:** 10.1371/journal.pone.0343621

**Published:** 2026-04-08

**Authors:** Akinkunle Oye-Somefun, Parmis Mirzadeh, Jenny Gao-Kang, Michael Rotondi, Jennifer L. Kuk, Hala Tamim, Chris I. Ardern

**Affiliations:** York University, Toronto, Ontario, Canada; University of Stirling, UNITED KINGDOM OF GREAT BRITAIN AND NORTHERN IRELAND

## Abstract

**Objective:**

This study aimed to summarize the observational evidence from prospective cohort studies examining the associations of regular physical activity, sedentary behaviour, and sleep duration with incident dementia among community-dwelling adults aged 35 years and older.

**Methods:**

Systematic literature searches (1946 to August 2025) of CINAHL, EMBASE, MEDLINE, PSYCINFO, and SPORTDISCUS were performed. Eligible studies included community-dwelling adults aged 35 + years with at least one year of follow-up and valid measures of movement behaviours and dementia outcomes. Studies were excluded if they included participants with baseline dementia, lacked risk estimates for all-cause dementia. Grey literature was excluded. Random effects meta-analysis generated pooled risk ratio (RR) and 95% confidence intervals (CI). Primary exposures were defined using national thresholds for physical activity, sedentary time, and sleep duration. Subgroup analyses were performed by age and follow-up duration.

**Results:**

Forty-nine studies with physical activity (n = 2,855,529), 17 studies on sleep duration (n = 1,344,170), and three studies on sedentary duration (n = 295,809) were included. Regular physical activity significantly reduced the risk of incident dementia (pooled RR = 0.75, 95% CI = 0.68 to 0.82), though heterogeneity was substantial and partially explained by subgroup analyses. Prolonged sedentary behaviour (8 + hours/day sitting) increased dementia risk (RR = 1.27, 95% CI = 1.17 to 1.39) with low heterogeneity. Moreover, both short (<7 hours; RR = 1.18, 95% CI = 1.09 to 1.28) and long (>8 hours; RR = 1.28, 95% CI = 1.15 to 1.43) sleep were linked to higher dementia risk compared with 7–8 hours. Heterogeneity was moderate to substantial.

**Conclusion:**

Regular physical activity, less sedentary time and appropriate nightly sleep (7–8 h) may be associated with reduced risk of dementia and are potentially modifiable factors in the prevention or delay of dementia. Future studies with middle-aged adults and longer-term follow-up including changes in movement behaviours over time are needed to better understand the relationship between physical activity, sedentary behaviour, and sleep for dementia risk.

**PROSPERO Registration:**

CRD42021272054.

## Introduction

Dementia refers to a group of neurodegenerative disorders characterized by cognitive decline and memory loss, and it is a leading cause of death in older adults [1]. Among these disorders, Alzheimer’s disease (AD) is the most common form, accounting for approximately 60–80% of all cases of dementia, with about 10 million new diagnoses of AD each year [2]. Currently, an estimated 55 million people worldwide are living with dementia [3]. This number is projected to triple by 2050, with approximately 60% of cases (dementia) occurring in low- and middle-income countries [4]. In 2018, the global cost of dementia care (direct and indirect expenses) was estimated at $1 trillion, and this figure is expected to double by 2030 [4–5].

Dementia results in severe worsening of cognitive function, disability, dependency, quality of life, and risk of death in individuals aged 65 years and over [6]. The preclinical or prodromal period of dementia may occur decades before symptoms [7–8], and available pharmacological interventions have provided only limited success at preventing or treating dementia [9], despite the use of a wide range of medications with adverse side effects [10]. Research on modifiable risk factors, including physical activity (PA), is important for informing evidence-based prevention strategies [11–13]. Older adults transitioning to retirement have low levels of regular physical activity [14], making this a period for accelerated health decline. Healthy movement behaviours, such as regular physical activity, limiting sedentary time, and appropriate nightly sleep, may be important for the management of dementia risk factors.

Physical activity has been linked with cognitive performance [15]; however, the relationship with dementia is less clear [16]. Epidemiological studies have shown that regular movement behaviours could lower the risk of cardiovascular disease including chronic inflammation [17–19] and are effective at lowering the risk of dementia and cognitive decline in older adult populations [20]. Sedentary behaviour is also a risk factor for cardiovascular disease. Prolonged sitting has been linked to a wide range of problems including insulin resistance, inflammation, oxidative stress, which present an increased risk of neurodegenerative disease [21]. Prolonged sedentary time may contribute to poor cognitive functioning and increased risk of cardiovascular disease [21–24]. Finally, sleep problems have also been associated with an increased risk of death [25], and dementia [26–27]; however, the effects of sleep duration and long-term sleep problems on the risk of dementia are not well known [28–29]. Because movement behaviours are independently related to cardiovascular health [21,30], it is important to understand how these movement behaviours work together to maintain brain health [31].

The risk of developing dementia tends to accelerate in older age [32], and it has been reported that the management of risk factors through lifestyle intervention could delay or prevent up to 45% of cases of dementia worldwide [33]. The 2024 Lancet Commission update highlights midlife physical inactivity as a modifiable risk factor, accounting for approximately 2% of new dementia cases, underscoring the importance of movement behaviours earlier in adulthood [33]

Understanding the relationship between movements behaviours and dementia risk at an earlier age is needed to help inform risk reduction strategies in susceptible middle-aged and older-aged adult populations. Community-dwelling adults represent the primary target population for preventive strategies, as lifestyle behaviours are largely self-directed and modifiable prior to the onset of severe cognitive impairment. Moreover, few systematic reviews and meta-analyses have examined these relationships in community-dwelling adult populations across diverse ages [34–35] and follow-up durations [36–37]. From this framework, we evaluated the relationships between established or recommended physical activity levels, sedentary behaviour, and sleep duration with incident dementia in community-dwelling adults, using a systematic review and meta-analysis of observational, prospective cohort studies.

## Methods

This systematic review and meta-analysis (SRMA) was registered in PROSPERO CRD42021272054. Systematic literature searches were conducted from the earliest available date in each database through August 2025 (MEDLINE 1946, EMBASE 1974, APA PsycInfo 1967, CINAHL 1994, SPORTDiscus 1992) using the standard online interfaces of each database without specialized search software or language restrictions, to identify relevant peer-reviewed journal articles related to reports of movement behaviours with dementia in community-dwelling adult populations. Search terms included (but were not limited to) the following: physical activity, walking, sedentary behaviour, sleep, memory, cognition, dementia, alone or combined using Boolean operators in each database (see S1 Table for the full electronic MEDLINE search strategy). Grey literature was not considered. Duplicate records were manually identified and removed during screening. References of retrieved articles were examined to locate additional relevant articles, and published studies were screened by title and abstract. Two independent reviewers (JGK and PM) screened studies by title and abstract and evaluated full-text articles for eligibility and study quality. Duplicate records were manually identified and removed during the screening process. Where necessary, consensus with a third reviewer (CIA) was used to resolve discrepancies. Risk of bias was evaluated using the risk of bias in non-randomized studies of interventions (ROBINS-I tool) [38]; the assessment was performed by a single reviewer (PM). To mitigate potential biases and enhance the reliability of the evaluation, the assessments were validated by a second reviewer (CIA).

Studies with appropriate definitions of incident dementia were included if their longitudinal cohort study designs involved measuring the outcome at follow-up. Longitudinal designs were defined a priori as those establishing temporal ordering between baseline exposure assessment and subsequent incident dementia, thereby minimizing reverse causation. Studies were eligible for inclusion if they: i) used a longitudinal cohort design; ii) included healthy, community-dwelling adults aged ≥35 years at baseline; and iii) assessed incident (new-onset) all-cause dementia during follow-up. Studies were excluded if they: i) lacked a valid measure of a primary exposure or outcome of interest; ii) had a follow-up period of less than 1 year; iii) included participants with a clinical diagnosis of dementia at baseline; iv) did not report a risk estimate for all-cause dementia at follow-up; or v) represented duplicate analyses of the same cohort participants as other included studies. In these instances, studies with larger samples over subsets, higher quality and more interpretable measures of physical activity levels, and longer follow-up times were prioritized to enhance generalizability. Analysis of data and presentation of results were based on the preferred reporting items for systematic reviews and meta-analyses (PRISMA) guidelines [39]. Ethics approval was not required from our host institution as there was no potential for participant identification from published summary data.

### Risk of bias assessment

We assessed risk of bias, and representativeness of the current evidence. A comprehensive score of bias was assigned by aggregating sources of bias using the Risk of Bias in Non-randomised Studies – of Interventions or “ROBINS-I Assessment Tool” for observational study designs [38,40]. Evaluation of study bias was done across seven domains, including: risk of bias due to confounding, participant recruitment and selection, classification to (or deviation from) assigned interventions, missing data, measurement, and selection of the reported result. Overall bias was subsequently classified as “Low”, “Moderate”, or “Serious” as per the open-source ROBINS-I risk aggregation tool (.xls) developed by Marcolino (2020).

### Outcome

The primary study endpoint was incident all-cause dementia according to neuropsychiatric evaluations, health registry data, and standardized clinical diagnostic criteria. We included studies where dementia was ascertained with or without dementia as the cause of death. Relevant data were extracted from individual studies including the most saturated (i.e., fully adjusted) primary outcome estimate (odds ratio (OR), risk ratio (RR), and hazard ratio (HR)) along with their 95% confidence intervals (CI) using a pre-specified format.

### Exposures

The primary study exposures were based on self-report questionnaire and operationalized to approximate established movement behaviour thresholds used by national agencies [41]. For physical activity, the “active” group was defined as participating in 150 minutes or more per week of aerobic activity and compared to the “inactive” group (or approximations thereof in comparing the highest vs lowest active groups in a distribution) [41]. For sedentary time, the optimal time spent sitting per day was defined as engaging in fewer than 8 hours of sitting, compared to prolonged sitting (8 + hours) [41]. For nightly sleep duration, shorter (<7 hours) and longer (>8 hours) sleep categories were compared to 7–8 hours of sleep per night [41].

### Statistical analyses

For each of our primary outcomes (PA, sedentary time and sleep), the primary statistical analyses pool effect measures from each individual study and their respective estimate of variance in a meta-analytic framework. We combined results of longitudinal cohort studies, including risk estimates that were adjusted for multiple confounding factors. Studies were assumed to be heterogeneous and random effects models were used to calculate pooled estimates (OR/RR/HR) under the rare disease assumption [42]. Risk estimates were log transformed for analysis purposes. The *I*^2^ statistic was used to assess the heterogeneity between studies [42]. Subgroup analyses were performed by age and follow-up duration. Forest plots were generated to illustrate the results of the random-effects meta-analysis, displaying both 95% CI and 95% prediction intervals (PI). A result was considered statistically significant when the 95% CI did not include 1 (null). In contrast, a 95% PI crossing 1 indicates potential between-study variability in future studies. Funnel plots were used to assess potential bias due to missing results (publication bias) for the overall analyses. The analysis was performed using R (version 4.1.3) [43] and the **metafor** package (version 3.4.0) [44] with a two-sided type 1 error rate of 5%.

## Results

**Fig 1:**
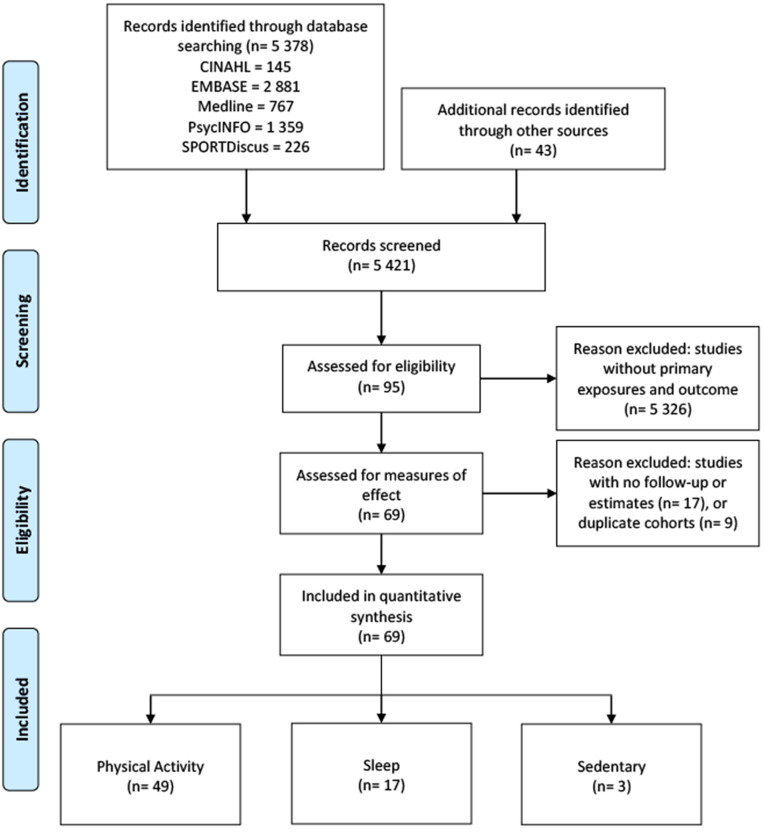
PRISMA flow diagram. Illustrates the study selection process..

The PRISMA flow diagram (see Fig 1) summarizes the studies selected for inclusion in this report consistent with the PRISMA statement [39]. Descriptive characteristics of each of the included studies are reported (see S2–S4 Tables). Reporting of variability (e.g., standard deviations) was inconsistent across studies and therefore is not shown.

### Quantitative synthesis

**Fig 2:**
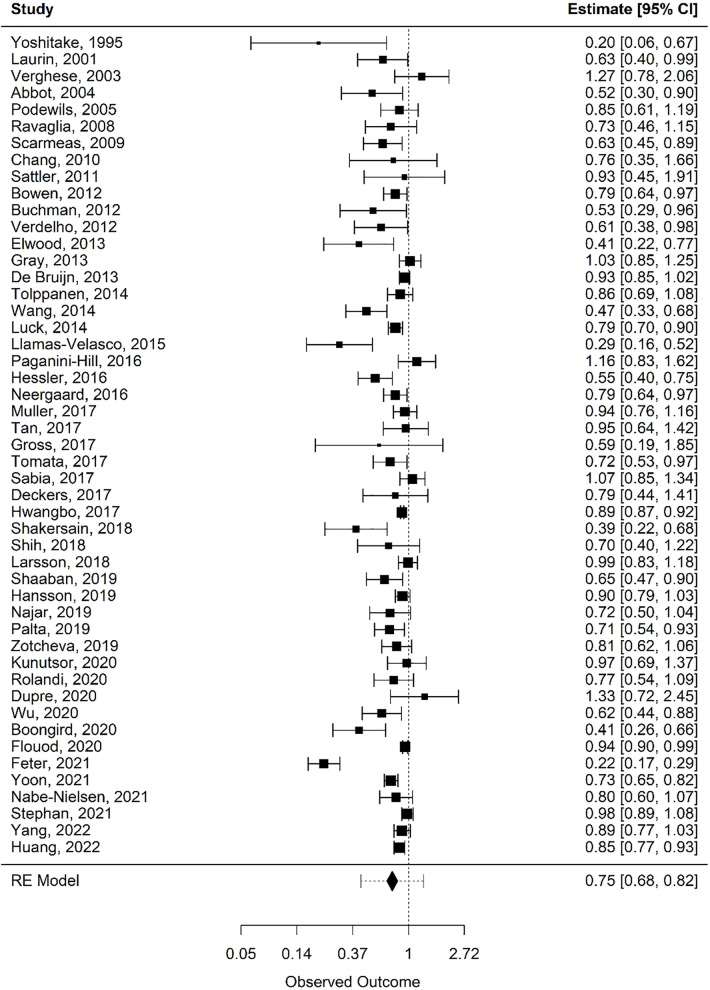
Forest plot: physical activity. Random-effects meta-analysis for associations between physical activity and incident dementia.

**Fig 3:**
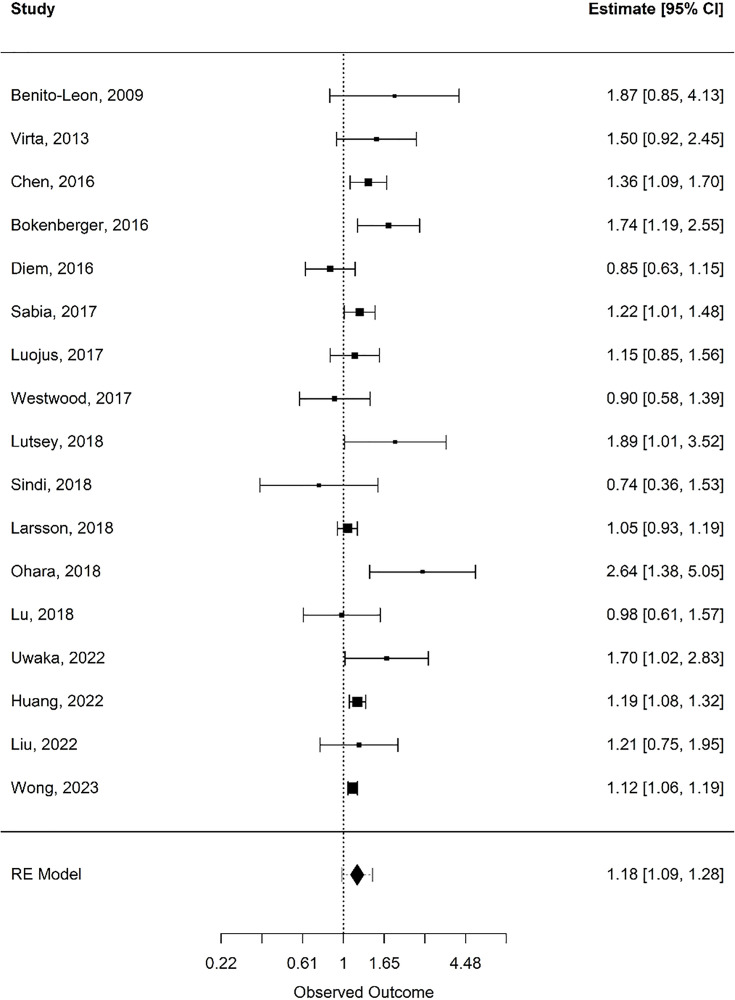
Forest plot: short sleep duration. Random-effects meta-analysis for associations between short sleep duration and incident dementia.

**Fig 4:**
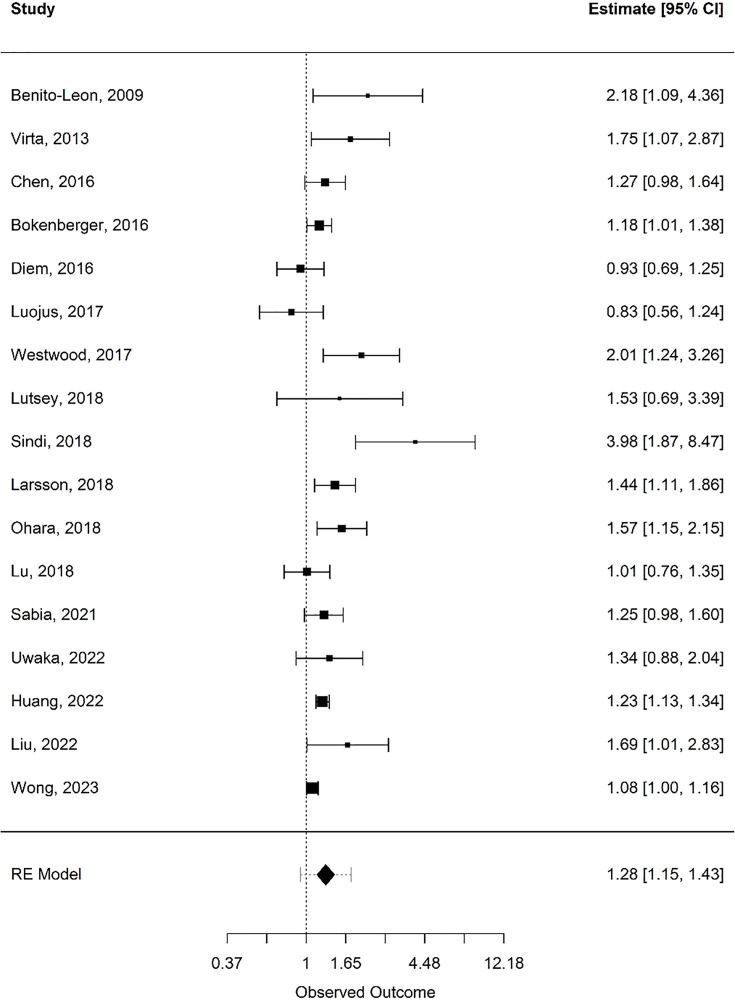
Forest plot: long sleep duration. Random-effects meta-analysis for associations between long sleep duration and incident dementia.

**Fig 5:**
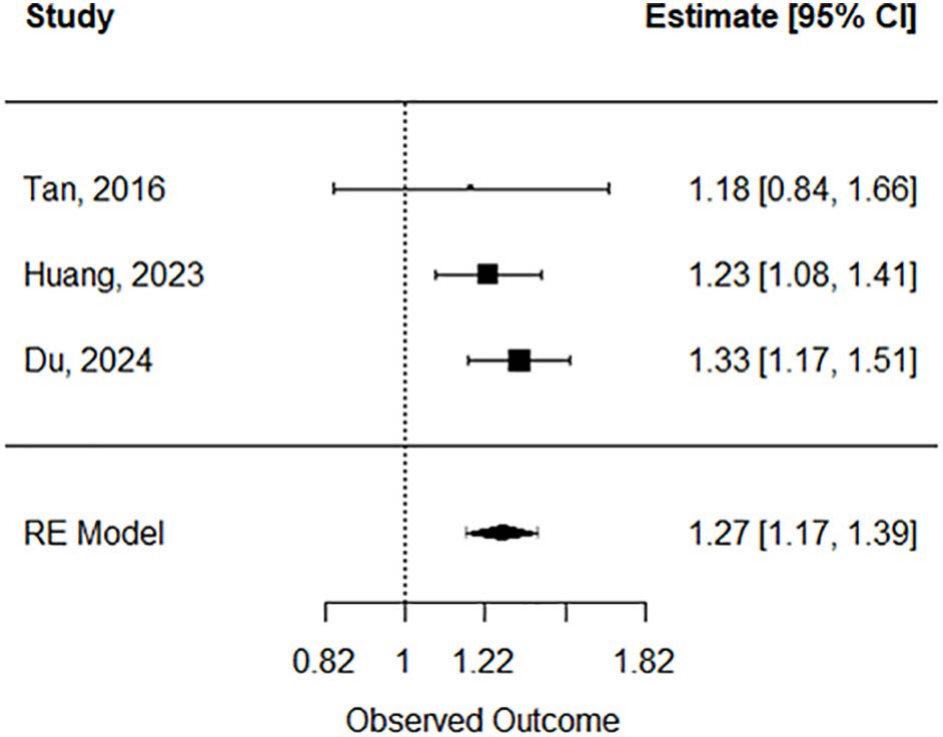
Forest plot: sedentary behaviour. Random-effects model meta-analysis for associations between sedentary behaviour and incident dementia.

Forest plots of the pooled estimates for analysis are included by movement behaviour type: physical activity (see Fig 2), sleep (see Fig 3 and 4), and sedentary time (see Fig 5). Funnel plots of the included studies are included by movement behaviour type: physical activity (see S5 Fig), sleep (see S6 and S7 Fig), and sedentary time (see S8 Fig).

### Physical activity

A total of 49 articles met the inclusion criteria for quantitative synthesis as it relates to physical activity [45–93]. Collectively, these studies included 2,855,529 participants, of whom 97,167 (3.4%) were diagnosed with dementia during follow-up. The mean age across studies was 67.3 years, and the mean follow-up duration was 11.6 years. Overall, 25 of the 49 studies (51.0%) included in the meta-analysis reported a statistically significant reduction in risk of dementia in the physically active middle-age and older adults. The overall (k = 49) random effects log risk ratio was calculated as −0.29 (95% CI = −0.39 to −0.20). The corresponding random effects risk ratio was 0.75 (95% CI = 0.68 to 0.82), which shows a statistically significant overall lower risk of dementia in middle-age and older adults that engaged in regular (i.e., moderate-vigorous intensity) physical activity compared to their non-active counterparts (Table 1). However, total heterogeneity between the studies was high (*I*^2^ = 92.5%). To explore this further, subgroup analysis was performed using the prespecified subgroups of age and follow-up duration to assess heterogeneity. The pattern of associations remained consistent while heterogeneity was reduced in the sub-group analyses (Table 1), but it does remain high suggesting other potential sources of heterogeneity.

**Table 1:** Meta-Analysis of Physical Activity studies. Associations between physical activity and incident dementia.

Outcomes	No. of studies	Effect estimate (RR)	95% CI	*I*^2^ (%)
***Main analysis***				
Physical Activity	49	0.75	0.68 to 0.82	92.5
***Subgroup analyses***				
Aged 35–64 years	20	0.76	0.64 to 0.89	96.9
65 years and older	29	0.74	0.66 to 0.82	73.9
Short follow-up duration (under 10 years	29	0.72	0.65 to 0.81	79.2
Long follow-up duration (10 years and over)	20	0.78	0.67 to 0.92	86.2
Aged 35–64 years old with short follow-up (under 10 years)	4	0.75	0.57 to 0.98	86.3
Aged 35–64 years old with long follow-up (10 years and over)	16	0.76	0.63 to 0.93	97.4
65 years and older with short follow-up (under 10 years)	25	0.72	0.63 to 0.81	76.8
65 years and older with long follow-up (10 years and over)	4	0.89	0.75 to 1.06	28.4

Abbreviations: RR, risk ratio; CI, confidence interval.

### Sleep

A total of 17 articles met the inclusion criteria for quantitative synthesis as it relates to sleep [76,93–103,104–108]. Together, these studies included 1,344,170 participants, of whom 49,581 (3.7%) were diagnosed with dementia at follow-up. The mean age across studies was 67.5 years, and the mean follow-up duration was 12.1 years. All 17 studies examined both short (< 7 hours) and long (> 8 hours) nightly sleep durations in relation to dementia risk, each comparing these to optimal sleep (7–8 hours per night). Across these studies, eight (47.1%) reported a statistically significant higher risk of dementia among short sleepers, and nine (52.9%) reported a higher risk among long sleepers. The random effects model showed a statistically significant risk of dementia for short (< 7 hours) sleepers (k = 17; log risk ratio = 0.17, 0.09 to 0.25; risk ratio = 1.18, 1.09 to 1.28; *I*^2^ **=** 41.1%), and long (> 8 hours) sleepers (k = 17; log risk ratio = 0.25, 0.14 to 0.36; risk ratio = 1.28, 1.15 to 1.43; *I*^2^ **=** 66.9%), compared to regular sleepers (7–8 hours of sleep). Subgroup analysis was performed with age and follow-up duration to assess heterogeneity in short and long sleepers (see Table 2). Overall heterogeneity in sleep study results was moderate to substantial but was lowest in studies of participants aged 35–64 years with follow-up periods ≥10 years.

**Table 2:** Meta-Analysis of Sleep studies. Associations between sleep duration and incident dementia.

Outcomes	No. of studies	Effect estimate (RR)	95% CI	*I*^2^ (%)
***Main analysis***				
Short Sleepers	17	1.18	1.09 to 1.28	41.1
Long Sleepers		1.28	1.15 to 1.43	66.9
			
***Subgroup analyses: Short Sleepers***				
Aged 35–64 years^*a*^	5	1.17	1.06 to 1.29	17.4
				
65 years and older	12	1.20	1.03 to 1.39	64.1
				
Short follow-up duration (under 10 years)*^b^*	8	1.20	0.98 to 1.46	59.8
				
Long follow-up duration (10 years and over)	9	1.19	1.07 to 1.32	45.5
				
Aged 35–64 years with long follow-up (10 years and over)^*a*^	5	1.17	1.06 to 1.29	17.4
65 years and older with short follow-up (under 10 years)^*b*^	8	1.20	0.98 to 1.46	59.8
65 years and older with long follow-up (10 years and over)	4	1.22	0.92 to 1.63	66.0
***Subgroup analyses: Long Sleepers***				
Aged 35–64 years^*c*^	5	1.09	1.02 to 1.18	1.1
				
65 years and older	12	1.39	1.19 to 1.62	72.2
				
Short follow-up duration (under 10 years)^*d*^	8	1.39	1.10 to 1.76	80.1
Long follow-up duration (10 years and over)	9	1.25	1.09 to 1.42	55.5
Aged 35–64 years with long follow-up (10 years and over)^*c*^	5	1.09	1.02 to 1.18	1.1
65 years and older with short follow-up (under 10 years)*^d^*	8	1.39	1.1 to 1.76	80.1
65 years and older with long follow-up (10 years and over)	4	1.44	1.15 to 1.82	55.7

Abbreviations: RR, risk ratio; CI, confidence interval

^a–b)^ Short Sleepers: Ages 35–64 and ages 35–64 with ≥10 years’ follow-up, and short follow-up (<10 years) and ages ≥65 with short follow-up, comprise the same underlying study populations; results are identical and reported in both rows for completeness.

^c–d)^ Long Sleepers: Ages 35–64 and ages 35–64 with ≥10 years’ follow-up, and short follow-up (<10 years) and ages ≥65 with short follow-up, comprise the same underlying study populations; results are identical and reported in both rows for completeness.

### Sedentary

In terms of sedentary duration, only three studies [68,109,110] met the criteria for quantitative synthesis. These studies followed 295,809 participants in total, of whom 6,212 (2.1%) were diagnosed with dementia. Participants had a mean age of 66.5 years, and the average follow-up period was 7.6 years. Two out of three (66.7%) studies with prolonged sitting time (8 + hours of daily sitting) reported a statistically significant higher risk of dementia compared to less sitting time (< 8 hours of daily sitting). The random effects model revealed a statistically significant higher risk of dementia for prolonged sitting time (k = 3; log risk ratio = 0.24, 0.15 to 0.33; risk ratio = 1.27, 1.17 to 1.39; *I*^2^ **=** 0%) (see Table 3).

**Table 3:** Meta-Analysis of Sedentary Behaviour studies. Associations between sedentary behaviour and incident dementia.

Outcomes	No. of studies	Effect estimate (RR)	95% CI	*I*^2^ (%)
***Main analysis***				
Sedentary Behaviour	3	1.27	1.17 to 1.39	0

Abbreviations: RR, risk ratio; CI, confidence interval.

Note: Although heterogeneity was estimated as 0%, the small number of studies limits the ability to detect variability; results should therefore be interpreted with caution.

### Risk of bias

ROBINS Risk of Bias assessment (**S9****–****S11 Tables**) demonstrated moderate risk of bias for the majority of physical activity studies (low: n = 3 (6.1%); moderate: n = 34 (69.4%), and serious: n = 12 (24.5%) out of 49 studies), and sleep studies (low: n = 1 (5.9%); moderate: n = 12 (70.6%), and serious: n = 4 (23.5%) out of 17 studies), and there was a serious risk of bias for the majority of sedentary studies (moderate, n = 1 (33.3%), and serious, n = 2 (66.7%) out of three studies). When present, the primary risk of moderate or serious bias was the result of bias due to confounding, followed by bias due to missing data. Common confounding variables included demographic factors (age, sex, education), genetic risk factors (e.g., apolipoprotein E (APOE) ε4 status), vascular or metabolic comorbidities (hypertension, diabetes, cardiovascular disease), baseline cognitive function, lifestyle behaviours (smoking, alcohol, BMI), and psychosocial factors (depression, social support). Most studies showed low risk of bias across selection, intervention, outcome measurement, and reporting domains, whereas the greatest variability was found for bias due to confounding. Measurement of physical activity, sleep, and dementia classification were the larger contributors to overall risk of bias, with both mixed outcome studies (e.g., Mild cognitive impairment (MCI)/AD/dementia) or more fitness-based assessment of physical activity showing increased risk of bias.

## Discussion

Results of this SRMA showed that adhering to established or recommended physical activity (PA) levels was associated with significantly lower incidence (25% reduced risk) of all-cause dementia; however, considerable heterogeneity in the random effects model for physical activity suggests unexplained variability. While we have investigated potential sources including baseline age differences, and follow-up duration, as well as controlling for potential confounders using adjusted models, additional sources of heterogeneity cannot be dismissed. Our SRMA also found that either short or long nightly sleep duration was associated with a 18% and 28% higher risk of dementia, respectively, compared to recommended 7–8-hour levels, with only moderate or substantial heterogeneity between studies. Finally, prolonged sedentary time was associated with a 27% higher risk of dementia, compared to recommended level (i.e., < 8 hours), and heterogeneity between studies was low.

These results are consistent with previous meta-analyses that showed an association between physical activity and all-cause dementia, including a 38% reduced risk in the most physically active, compared to less active counterparts [34–36,111]. However, our SRMA expands these findings with more recent studies from larger, diverse populations (primarily from high-income countries, with variation in age, sex, and, where reported, race/ethnicity), with longer follow-up times, and by focusing on middle- and older-age adults more broadly. Although heterogeneity was moderate-to-substantial, physical activity studies with older baseline ages and/or shorter follow-up periods had less heterogeneity. This is expected considering the challenges with long-term tracking of physical activity in older populations. As such, many studies examining physical activity in older age tend to have follow-up durations of less than 10 years [112–114]. Furthermore, our SRMA included studies with adults aged 35–64 years at baseline; however, heterogeneity was relatively high, which also increased with long-term tracking.

In the studies included in our sleep analysis, sleep duration was consistently categorized into short, regular, and long nightly sleep, with regular sleep (7–8 hours) as the reference category. Risk estimates were reported for both short and long sleep within the same study. While a recent meta-analysis found that only long sleep duration was associated with dementia [115], our study showed that nightly sleep both below and beyond 7–8 hours was associated with higher incidence of all-cause dementia, and the risk was higher with long sleep duration. These findings align with prior research examining sleep and broader cognitive outcomes [96,102,116]. Of note, few longitudinal studies investigated the relationship between regular sleep and incident dementia [117], and future studies involving midlife and longer follow-ups are needed to better ascertain these relationships [106,118,119].

Prolonged sedentary behaviour is associated with poorer physical health, including obesity, diabetes, and cardiovascular disease [21]. These conditions are associated with reduced physical functioning and increase limitations in daily tasks needed to care for oneself and live independently, which are known dementia risk factors [120]. Although the search strategy was comprehensive, several identified records (n = 4) did not explicitly investigate or adequately describe sedentary behaviour [65,121–123], limiting their inclusion in quantitative synthesis. Nevertheless, a small number of longitudinal studies demonstrate associations between sedentary time and dementia risk. Across these studies, longer sedentary duration was associated with higher risk of dementia. Sedentary behaviour recently began to be recognized as a distinct modifiable behaviour around 2012 [124,125]. As a result, several large cohort studies did not capture detailed or validated measures of sedentary behaviour [124]. Moreover, in some studies, low physical activity was characterized as sedentary behaviour though they are now recognized as distinct and different behaviours as opposed to two ends of one continuous spectrum [125,126,127]. Although prolonged sedentary time might have adverse independent effects on brain health such as cognitive decline, or mild cognitive impairment [128], evidence regarding incident dementia as a clinical endpoint remained limited, largely due to insufficient follow-up periods and the relative scarcity of high-quality sedentary behaviour data.

Lifestyle modifications are common public health interventions that could have durable effects in the prevention and delay of dementia [129,130]. Although the potential mechanisms and pathways are not fully understood [128], increased physical activity with reduced sedentary time has been shown to improve physical function [129], and quality of life in older adults [131,132]. Indeed, regular physical activity stimulates antioxidative (protective) processes, which enhance blood flow, brain vasculature, and hippocampal volume [133]. Regular physical activity has also been found to increase brain-derived neurotrophic factor (BDNF) and insulin-like growth factor (IGF-1), which confer neuroprotection and neurotransmitter integrity [134,135]. Importantly, the proposed mechanisms of physical activity, sedentary time, and sleep on brain health are not mutually exclusive, and may act through, or on other established chronic disease risk factors [136,137]. In the sleep-dementia relationship, the glymphatic system is most active during sleep to regulate inflammation in the brain [136,137], and the process of brain aging may be accelerated by suboptimal sleep [136]. When coupled with physical inactivity, sleep disruption can exacerbate the atrophy of brain structures [138]. Although the mechanisms underlying long sleep are less well understood, studies suggest potential roles for underlying comorbidities, systemic inflammation, or early neurodegenerative changes [115,135]. Taken together, a healthy movement behaviour profile may promote vascular, neurotrophic, and anti-inflammatory effects that collectively support brain health and may delay the onset or progression of dementia.

### Strengths and limitations

Strengths of this study include: i) valid and reliable risk estimates of dementia using clinical diagnoses, ii) pooling of large community-dwelling population-based cohorts with long-term follow-ups increased statistical power to detect these effects, iii) the breadth of countries and diverse populations, which allowed for a robust analysis of these measures and generalizability of results, and iv) assessment of publication bias using visual inspection of the funnel plot. Nonetheless, our approach also had some limitations. First, heterogeneity across physical activity studies requires further investigation into potential unexplained factors. Second, while reverse causation is unlikely given the exclusion of participants with dementia at baseline, it cannot be entirely ruled out in studies with short follow-up periods. In such cases, participants may already have early symptoms of dementia that were not formally diagnosis. Physical activity, sedentary behaviours, and sleep were self-reported, and sleep *quality* was not accounted for; therefore, interpretations of results should include careful consideration of recall bias. Objective measures (e.g., wearable devices) of physical activity, sedentary time, and sleep could enhance internal validity of study findings by providing timely, accurate, and detailed understanding of movement behaviours in middle- and older-age adults with limitations in physical fitness and recall. However, there is no universally accepted standard for measuring these behaviours, and different types of objective measures may yield varying results [124]. Comparison of our self-report findings should therefore be compared to a future SRMA with objective measures, as their use in cohort studies becomes more common.

The presence of moderate or serious risk of bias across studies (particularly due to confounding and missing data) has important implications for interpretation of our findings. Of note residual and unmeasured confounding (e.g., pre-existing health conditions, functional status, socioeconomic status, and health behaviours correlated with movement behaviours) may have inflated or attenuated observed associations. In addition, differential loss to follow-up and incomplete outcome ascertainment could have introduced selection bias. Taken together, these types of bias preclude causal inference and mean that our pooled estimates should be interpreted as associations rather than causal effects. Future studies would benefit from repeated measures of movement behaviours, and improved retention to reduce missing data and strengthen causal inference.

Although most studies accounted for key variables such as age, sex, and education, fewer studies additionally adjusted for comorbidities or lifestyle factors. Future studies could further mitigate confounding by comprehensive baseline assessment and statistical adjustment of relevant variables. Further sensitivity analyses to assess the robustness of findings, for example by adjusting for additional covariates or examining subgroups (e.g., sex, age, genetic risk), would strengthen the literature base overall. Finally, future studies should also aim to include participants from underrepresented populations, with particular emphasis on low- and middle-income countries, to broaden the geographic and socioeconomic representation of research. Given the demographics of the existing pool of studies, variability across studies should be interpreted in the context of broader social determinants of health and structural inequities.

Given that dementia could precede a clinical diagnosis by a decade [7], generalizability of evidence-based research on modifiable risk factors of dementia is a notable challenge [11]. Dementia has a long preclinical or prodromal period [7,8], which contributes to variability in the time to diagnosis of dementia. In the study of physical activity and sleep with dementia, this could contribute to reverse causation, particularly in light of our reliance on self-reported (behavioural) measures of physical activity and sleep [139]; however, decreases in physical activity across time would bias our results to the null. Our meta-analysis considered age (35–64 or 65 + y) and follow-up (<10 or 10 + y), and results did not change substantially; however, heterogeneity between studies reinforced the influence of other unmeasured factors. Previous research reported significant results with short (below 10 years) follow-ups [36,37] or in older (65 + years) populations [34,35]. Across all included studies, sex-specific analyses were rare. Some cohorts included only females or only males, and very few studies reported results stratified by sex. As a result, our ability to examine sex differences in dementia risk was limited. Indeed, building on previous meta-analyses with incident dementia [36,111,115], more studies with mid-life changes in physical activity, sedentary time, and sleep, and longer follow-ups are needed to ascertain the effects of changes in physical activity and sleep on dementia. Future studies, including compositional analyses to examine how reallocating time across movement behaviours influences cognitive decline and the development of dementia, are needed to further clarify these associations [41]. To enhance representation, studies should include diverse cohorts and present results disaggregated by sex to provide a clearer understanding of sex differences.

Findings from this review highlight opportunities for tailored public health strategies to support brain health in middle- and older-age adults. Technology-assisted tools (such as wearable activity trackers or smartphone apps) can help monitor and encourage physical activity, reduce sedentary behaviour, and support healthy routines. The use of personalized interactive prompts (based on past activity and personal interests) within these digital tools represents an emerging area of research that may support short-term uptake and facilitate longer-term behaviour change. Community programs (such as group exercise classes, walking groups, or educational activities related to sleep) may provide structured opportunities for movement, while broader policy initiatives, including accessible green spaces and workplace practices that reduce prolonged sitting, may support population-level approaches to healthier daily routines.

## Conclusion

Our study shows that the physical activity, increased sedentary time, and short or long sleep duration outside the recommended range were associated with increased risk of dementia. Future studies with longer-term follow-up and larger sample sizes to address issues of dose-response and alterability in the movement behaviour-dementia relationships are needed to better understand these relationships. Taken together, our findings have important clinical implications that could inform guidelines across the life course, including implementation of tailored public health strategies. Whether changes in physical activity, sleep, or sedentary behaviour – a construct distinct from physical inactivity – is also related to dementia, and whether these associations are independent of risk factors such as pre-existing health risks, remain areas in need of future work.

## Supporting information

S1 Table Search strategy example. MEDLINE search terms and results.
(PDF)

S2 Table Study characteristics: physical activity. Association between physical activity and dementia risk.
(PDF)

S3 Table Study characteristics: sleep duration. Association between sleep duration and dementia risk.
(PDF)

S4 Table Study characteristics: sedentary behaviour. Association between sedentary behaviour and dementia risk.
(PDF)

S5 Fig Funnel plot: physical activity. Graphical representation of publication bias for associations between physical activity and incident dementia.
(PDF)

S6 Fig Funnel plot: short sleep. Graphical representation of publication bias for associations between short sleep and incident dementia.
(PDF)

S7 Fig Funnel plot: long sleep. Graphical representation of publication bias for associations between long sleep and incident dementia.
(PDF)

S8 Fig Funnel plot: sedentary behaviour. Graphical representation of publication bias for associations between sedentary behaviour and incident dementia.
(PDF)

S9 Table Risk of bias assessment: physical activity. Association between physical activity and dementia.
(PDF)

S10 Table Risk of bias assessment: sleep duration. Association between sleep duration and dementia.
(PDF)

S11 Table Risk of bias assessment: sedentary time. Association between sedentary time and dementia.
(PDF)

S1 File PRISMA 2020 Checklist. (DOCX)
