# Supplementary material for: The Relationships between physical activity, sedentary behaviour, sleep, and dementia: A systematic review and meta-analysis of cohort studies
Source: PLoS One. 2026 Apr 8;21(4):e0343621. doi: 10.1371/journal.pone.0343621 (PMC13061222; doi:10.1371/journal.pone.0343621)
Supplement: S1 Table — MEDLINE search terms and results. (PDF) [file pone.0343621.s001.pdf]

S1 Table. Search strategy example. MEDLINE search terms and results.		
#	**Searches**	**Results**
1	Physical Activity.mp.	126263
2	exp Exercise/	224716
3	exp Exercise Movement Techniques/	9456
4	exp Exercise Therapy/	58083
5	Physical Exertion/	57156
6	Motor Activity/	99224
7	exp Sports/	201494
8	(sport$ or bicycl$ or swim$ or walk$ or run$ or jog$).tw.	473315
9	(physical$ adj2 activ$).tw.	133636
10	(aerobic adj2 (train$ or active$)).tw.	5291
11	or/1-10	881761
12	Sedentary Behaviour/	12085
13	Screen Time/	819
14	(sitting or sedentar$).tw.	36089
15	low energy expenditure.tw.	191
16	(computer game* or video game* or television or tv).tw.	31515
17	(physical* adj2 inactivit*).tw.	8982
18	(Reading/ or Books/) and ((time* or duration*).tw. or Time/ or Time Factors/)	6104
19	or/12-18	82840
20	Sleep/	60729
21	(sleep adj3 duration).tw.	11449
22	20 or 21	65849
23	(cognit* or dement*).tw.	517487
24	(cogn* adj3 assess*).tw.	27360
25	(cogn* adj3 dis*).tw.	38394
26	(cogn* adj3 eval*).tw.	9258
27	(cogn* adj3 funct*).tw.	86146
28	(cogn* adj3 imp*).tw.	114859
29	(cogn* adj3 meas*).tw.	18656
30	(memory adj3 imp*).tw.	41791
31	or/23-30	540546
32	(11 or 19 or 22) and 31	42769
33	limit 32 to humans	31561
34	limit 33 to adult	10047
35	exp Child/	2043362
36	exp Infant/	1204271
37	exp Adolescent/	2153069
38	exp Students/	149554
39	or/35-38	3900395
40	34 not 39	7182
41	cohort studies/ or follow-up studies/ or longitudinal studies/ or prospective studies/	1532217
42	longitudinal studies.mp.	171890

43	follow-up studies.mp.	689792
44	cohort studies.mp.	327817
45	prospective studies.mp.	644218
46	or/42-45	1603919
47	41 or 46	1603919
48	40 and 47	1008
49	clinical trial/ or controlled clinical trial/ or randomized controlled trial/ or clinical trial protocol/ or clinical trial, veterinary/	905707
50	48 not 49	767
Note: Although the search strategy was comprehensive, the records identified did not explicitly investigate or sufficiently describe sedentary behaviours for quantitative synthesis.		
