# Supplementary material for: The Relationships between physical activity, sedentary behaviour, sleep, and dementia: A systematic review and meta-analysis of cohort studies
Source: PLoS One. 2026 Apr 8;21(4):e0343621. doi: 10.1371/journal.pone.0343621 (PMC13061222; doi:10.1371/journal.pone.0343621)
Supplement: S2 Table — Association between physical activity and dementia risk. (PDF) [file pone.0343621.s002.pdf]

S2 Table. Study characteristics: physical activity. Association between physical activity and dementia risk.						
**Author and Country**	**Study and Sample**	**Design and Duration of Follow-up**	**Covariates**	**Physical Activity Measure**	**Incident Dementia**	**Risk Estimate (95%CI)**
Yoshitake et al., 1995 [45]<br><br>Japan	Hisayama study; n=828 (59.7% W, mean age: 73.6 y).	Prospective longitudinal design (average follow-up: 7 y).	Age, sex, physical activity, and Hasegawa's dementia scale.	Self-reported physical activity performed 3+ times per week.	Incident dementia cases (n=103) based on standard clinical evaluation criteria.	Highest PA level:<br><br>RR, 0.20, 0.06 – 0.68
Laurin et al., 2001 [46]<br><br>Canada	Canadian Study of Health and Aging; n=6,434 (60.3% W; mean age: 73.1 y).	Prospective longitudinal design (average follow-up period: 5 y).	Age, sex, and education.	Self-reported physical activity performed 3+ times per week.	Incident dementia cases (n=285) based on standard clinical evaluation criteria.	Highest PA level:<br><br>OR, 0.63, 0.4 – 0.98
Verghese et al., 2003 [47]<br><br>United States of America	Bronx Aging Study; n=469 (64.1% F; mean age: 79.1 y).	Prospective longitudinal design (average follow-up: 5.1 y).	Age, sex, education, chronic medical illness, baseline score on the Blessed Information-Memory-Concentration test.	Self-reported physical activity, based on a score (0-77) of 16+ points.	Incident dementia cases (n=124) based on standard clinical evaluation criteria.	Highest PA level:<br><br>HR, 1.27, 0.78 – 2.06
Abbot et al., 2004 [48]<br><br>United States of America	Honolulu-Asia Aging Study; n= 2,257 M (mean age: 76.8 y).	Prospective longitudinal design (average follow-up: 6 y).	Age, APOE, baseline Cognitive Abilities Screening Instrument score, decline in physical activity since mid adulthood, physical performance score, education, BMI, childhood years spent living in Japan, status as a skilled professional, hypertension, diabetes, prevalent coronary heart disease, and total and high-density lipoprotein cholesterol.	Self-reported physical activity at two time points, based on a physical activity index, resulting from the inverse of low PA.	Incident dementia cases (n=158) based on standard clinical evaluation criteria.	Highest PA level [i]:<br><br>HR, 0.52, 0.3 – 0.9
Podewils et al., 2005 [49]<br><br>United States of America	Cardiovascular Health Cognition Study (CHCS); n=3,375 (59.1% F, mean age: 74.8 y).	Prospective longitudinal design (average follow-up: 5.4 y).	Age, education, gender, ethnicity, APOE, baseline Modified Mini-Mental State Examination score, magnetic resonance imaging white-matter-grade score, activities of daily living impairment, instrumental activities of daily living	Self-reported physical activity based on the highest quartile resulting from metabolic equivalent of task (MET) per week.	Incident dementia cases (n=480) based on standard clinical evaluation criteria.	Highest PA level:<br><br>HR, 0.85, 0.61 – 1.19

			impairment, Lubben Social Network Score, and social support score.			
Ravaglia et al., 2008 [50]<br><br>Italy	Conselice Study of Brain Aging (CSBA); n=469 (53.5% W; mean age: 79.1 y).	Prospective longitudinal design (average follow-up: 3.9 y).	Age, gender, education, APOE, cardiovascular disease, hypertension, hyperhomo-cysteinemia, comorbidity, basic activities of daily living.	Self-reported physical activity, based on 150+ minutes per week of moderate-to-vigorous PA.	Incident dementia cases (n=86) based on standard clinical evaluation criteria.	Highest PA level:<br><br>HR, 0.73, 0.46 – 1.15
Scarmeas et al., 2009 [51]<br><br>United States of America	Washington Heights-Hamilton Heights-Inwood Community Aging Project (WHICAP); n=1,880 (69% F; mean age: 77.2 y).	Prospective longitudinal design (average follow-up: 5.4 y).	Age, sex, ethnicity, education, APOE, caloric intake, BMI, smoking, depression, leisure activities, comorbidity index, baseline Clinical Dementia Rating score, and time between first dietary and first physical activity assessment.	Self-reported physical activity resulting from a score categorized into tertiles.	Incident dementia cases (n=282) based on standard clinical evaluation criteria.	Highest PA level:<br><br>HR, 0.63, 0.45 – 0.9
Chang et al., 2010 [52]<br><br>Iceland	Reykjavik study; n=4,945 (57.7% F, mean age: 51.1 y).	Prospective longitudinal design (average follow-up: 25.7 y).	Age, sex, education, BMI, systolic blood pressure, smoking, and cholesterol.	Self-reported physical activity, performed 5+ hours per week.	Incident dementia cases (n=184) based on standard clinical evaluation criteria.	Highest PA level:<br><br>OR, 0.76, 0.34 – 1.63
Sattler et al., 2011 [53]<br><br>Germany	Interdisciplinary Longitudinal Study of Adult Development (ILSE); n=500 (49.3% F, mean age: 74.1 y).	Prospective longitudinal design (average follow-up: 12 y).	Age, gender, education, socioeconomic status, and depressive symptoms.	Self-reported physical activity performed 2+ hours per week.	Incident dementia cases (n=24) based on standard clinical evaluation criteria.	Highest PA level:<br><br>OR, 0.93, 0.45 – 1.9
Bowen et al., 2012 [54]<br><br>United States of America	Aging, Demographics, and Memory Study (ADAMS) from Health and Retirement Study (HRS); n=808 (58.5% F; mean age: 77.5 y).	Prospective longitudinal design (average follow-up: 5 y).	Age, sex, education, race, APOE, BMI, diabetes, hypertension, stroke, heart disease, smoking, and alcohol.	Self-reported physical activity performed 3+ times per week.	Incident dementia cases (n=277) based on standard clinical evaluation criteria.	Highest PA level:<br><br>OR, 0.79, 0.64 – 0.97

Buchman et al., 2012 [55]<br><br>United States of America	Rush Memory and Aging; n=716 (76% F, mean age: 81.6 y).	Prospective longitudinal design (average follow-up: 4 y).	Age, sex, and education, social, and cognitive activities, as well as current level of motor function, depressive symptoms, chronic health conditions, and APOE.	Physical activity was measured objectively using actigraphy.	Incident dementia cases (n=71) based on standard clinical evaluation criteria.	Highest PA level:<br><br>HR, 0.53, 0.29 – 0.95
Verdelho et al., 2012 [56]<br><br>Multi-centre European Countries	The Leukoaraiosis and Disability (LADIS) study; n=639 (55.0% F, mean age: 74.1 y).	Prospective longitudinal design (follow-up to 3 y).	N/A	Self-reported physical activity, 30 minutes of activity at least 3 days per week.	Incident dementia cases (n=90) based on standard clinical evaluation criteria.	Highest PA level:<br><br>HR, 0.61, 0.38 – 0.98
Luck et al., 2013 [57]<br><br>Germany	German study on Ageing, Cognition and Dementia in Primary Care Patients (AgeCoDe); n=2,492 (64.7% F, mean age: 81.1 y).	Prospective longitudinal design (average follow-up: 4.5 y).	Age, gender, education, alcohol consumption, smoking, Modified Mini-Mental State Examination score, mental activity, comorbidity (diabetes mellitus, hypertension, cardiac arrhythmia, coronary heart disease, myocardial infarction, peripheral arterial obstructive disease, carotid artery stenosis > 80%, transient ischaemic attack, stroke, hyperlipidemia, hypercholesterolaemia, hyperthyroidism, hypothyroidism, traumatic brain injury and depression), and APOE ε4 status.	Self-reported physical activity, performed 1+ times per week.	Incident dementia cases (n=278) based on standard clinical evaluation criteria.	Highest PA level:<br><br>HR, 0.79, 0.7 – 0.9
De Bruijn et al., 2013 [58]<br><br>Netherlands	The Rotterdam Study; n=4,406 (59.0% F, mean age: 72.7 y).	Prospective longitudinal design (average follow-up: 8.8 y).	Age, sex, Modified Mini-Mental State Examination score, low educational level, smoking, APOE-e4 carrier status, hypertension, BMI, diabetes, total cholesterol, and HDL cholesterol.	Self-reported physical activity, based on the metabolic equivalent of task (MET) hours.	Incident dementia cases (n=583) based on standard clinical evaluation criteria.	Highest PA level:<br><br>HR, 0.93, 0.85 – 1.02
Elwood et al., 2013 [59]	Caerphilly cohort study; n=2,235 M (mean age: 52 y).	Prospective longitudinal design (average follow-up: 30 y).	Age, social class, and National Adult Reading Test at baseline.	Self-reported physical activity performed 3+ times per week.	Incident dementia cases (n=79) based on standard	Highest PA level:<br><br>OR, 0.41, 0.22 – 0.77

United Kingdom					clinical evaluation criteria.	
Gray et al., 2013 [60] United States of America	Adult Changes in Thought (ACT) study; n=2,619 (60.1% F, mean age: 76.8 y).	Prospective longitudinal design (average follow-up: 6.5 y).	Age, sex, education, race, BMI, depressive symptoms, antidepressant use, self-reported health, hypertension, diabetes, myocardial infarction, congestive heart failure, smoking, and baseline Cognitive Abilities Screening Instrument score.	Self-reported physical activity, resulting from the inverse of low PA.	Incident dementia cases (n=521) based on standard clinical evaluation criteria.	Highest PA level [i]: HR, 1.03, 0.85 – 1.25
Tolppanen et al., 2014 [61] Finland	Cardiovascular Risk Factors, Aging and Dementia (CAIDE); n=1,511 (62.3% F, mean age: 50.6 y).	Prospective longitudinal design (average follow-up: 28.3 y).	Age, sex, education, midlife BMI, marital status, occupational physical activity level, smoking, and cardiorespiratory and musculoskeletal conditions.	Self-reported physical activity, resulting from the inverse of low PA.	Incident dementia cases (n=250) based on standard clinical evaluation criteria.	Highest PA level [i]: HR, 0.86, 0.69 – 1.08
Wang et al., 2014 [62] United States of America	Study of Osteoprotic Fractures (SOF); n=1,249 W (mean age: 83.3 y).	Prospective longitudinal design (average follow-up: 5 y).	Age, educational level, cognition, depressive symptoms, BMI, hypertension, smoking, and coronary artery disease.	Self-reported physical activity, resulting from the number of city-blocks or equivalent (10 blocks ≈ 1 mile) typically walked each day.	Incident dementia cases (n=212) based on standard clinical evaluation criteria.	Highest PA level: OR, 0.47, 0.33 – 0.69
Llamas-Velasco et al., 2015 [63] Spain	Neurological Disorders in Central Spain (NEDICES); n=3,105 (56.6% F, mean age: 73.2 y).	Prospective longitudinal design (follow-up to 3 y).	Age, sex, education, alcohol consumption, stroke, hypertension and Charlson Index.	Self-reported physical activity, high physical activity	Incident dementia cases (n=134) based on standard clinical evaluation criteria.	Highest PA level: HR, 0.29, 0.16 – 0.52
Neergaard et al., 2016 [64] Denmark	The Prospective Epidemiologic Risk Factor (PERF I) study; n=5,512 W (mean age: 70.5 y).	Prospective longitudinal design (follow-up: 11.9 y).	Age, education, BMI, smoking, alcohol, hypertension, history of cerebral embolism/hemorrhage, diabetes, history of depression, and history of other neural disorders.	Self-reported physical activity, 3+ times/week.	Incident dementia cases (n=592) based on standard clinical evaluation criteria.	Highest PA level: HR, 0.79, 0.64 – 0.97
Paganini-Hill et al., 2016 [65]	The 90+ study; n=587, (demographic data not available, mean age: 93 y).	Prospective longitudinal design (average follow-up: 3 y).	Age, sex, and education.	Self-reported physical activity performed 1+ times per week.	Incident dementia cases (n=268) based on standard clinical evaluation criteria.	Highest PA level: HR, 1.16, 0.83 – 1.62

United States of America						
Hessler et al., 2016 [66] Germany	Interventionsprojekt zerebrovaskuläre Erkrankungen und Demenz im Landkreis Ebersberg (INVADE-trial) study; n=3,547 (59.2% F, mean age: 67.3 y).	Prospective longitudinal design (average follow-up: 6.7 y).	Age, sex, and education.	Self-reported physical activity performed 3+ times per week, resulting from the inverse of physical inactivity.	Incident dementia cases (n=296) based on standard clinical evaluation criteria.	Highest PA level [i]: HR, 0.55, 0.41 – 0.76
Muller et al., 2017 [67] United States of America	Veterans Exercise Testing Study; n=6,104 (3.5% F, mean age: 59.2y).	Prospective longitudinal design (average follow-up: 10.3 y).	Unadjusted (did not include covariates).	Self-reported physical activity performed 3+ times per week, resulting from the inverse of physical inactivity.	Incident dementia cases (n=353) based on standard clinical evaluation criteria.	Highest PA level [i]: HR, 0.94, 0.76 – 1.16
Tan et al., 2017 [68] United States of America	Framingham Study Original and Offspring; n=3,714 (54.4% F; mean age: 70.5 y).	Prospective longitudinal design (average follow-up: 7.5 y).	Age, sex, high school degree, APOE *e4 allele status*, log plasma homocysteine, systolic blood pressure, diastolic blood pressure, antihypertensive medication, total cholesterol, current smoking, prevalent cardiovascular disease, diabetes, stroke, and atrial fibrillation.	Self-reported physical activity, based on the highest quantile (a score resulting from oxygen consumption or metabolic equivalents).	Incident dementia cases (n=236) based on standard clinical evaluation criteria.	Highest PA level: HR, 0.95, 0.63 – 1.41
Gross et al., 2017 [69] United States of America	Johns Hopkins Precursors study; n=646 (7.4% W, mean age: 47.1 y).	Prospective longitudinal design (average follow-up: 30 y).	Age, sex, smoking, diabetes, and hypertension.	Self-reported physical activity, based on metabolic equivalents (MET h/day).	Incident dementia cases (n=28) based on standard clinical evaluation criteria.	Highest PA level: HR, 0.59, 0.19 – 1.87
Tomata et al., 2017 [70] Japan	Ohsaki cohort study; n=6,909 (55.6% W, mean age: 74.6 y).	Prospective longitudinal design (average follow-up: 5.7 y).	Age, history of disease (stroke, hypertension, myocardial infarction, diabetes or hyperlipidemia), education, smoking, alcohol, psychological	Self-reported physical activity at a two time points; based on 1+ hours per day.	Incident dementia cases (n=638) based on standard clinical evaluation criteria.	Highest PA level: HR, 0.72, 0.53 – 0.97

			distress score, pain, and physical functioning level.			
Sabia et al., 2017 [71]<br><br>United Kingdom	Whitehall II study; n=10,308 (33.1% F, mean age: 45 y).	Prospective longitudinal design (average follow-up: 26.6 y).	Age, sex, ethnicity, education, occupational position, marital status, smoking status, alcohol consumption, and fruit and vegetable consumption, hypertension, diabetes, BMI, General Health Questionnaire score, cardiovascular disease, and cardiovascular disease drugs.	Self-reported physical activity, based on 2+ hours per week of moderate-to-vigorous PA.	Incident dementia cases (n=329) based on standard clinical evaluation criteria.	Highest PA level:<br><br>HR, 1.07, 0.86 – 1.35
Deckers et al., 2017 [72]<br><br>United Kingdom	Cambridge City over-75s cohort (CC75C) study; n=296 (68.0% F, mean age: 87.9 y).	Prospective longitudinal design (follow-up to 18 y).	Age, sex, educational level.	Self-reported physical activity, performed 1+ times per week.	Incident dementia cases or severe cognitive impairment (n=84) based on standard clinical evaluation criteria.	Highest PA level [i]:<br><br>OR, 1.28, 0.71 – 2.27
Hwangbo et al., 2017 [73]<br><br>South Korea	National Health Insurance Service (NHIS) data; n=794,448 (54.1% F, 58.5 y).	Prospective longitudinal design (follow-up: 12.0 y).	Sex, age, hypertension, diabetes, depression, osteoporosis, ischemic stroke, hyperlipidemia, coronary heart disease, smoking, and obesity.	Self-reported physical activity, >10 minutes of moderate-vigorous activity once a week.	Incident dementia cases (n=49,524) based on standard clinical evaluation criteria.	Highest PA level [i]:<br><br>RR, 0.89, 0.87 – 0.92
Shakersain et al., 2018 [74]<br><br>Sweden	Swedish National study on Aging and Care-Kungsholmen (SNAC-K); n=2,223 (60.8% W; mean age: 70.6 y).	Prospective longitudinal design (average follow-up: 6 y).	Age, sex, education, civil status, total calorie intake, dietary vitamin/mineral supplement use, smoking, BMI, vascular disorders, cancer, diabetes, depression, APOE, diet, and leisure activities.	Self-reported physical activity performed 3+ times per week.	Incident dementia cases (data not available) based on standard clinical evaluation criteria.	Highest PA level:<br><br>HR, 0.39, 0.22 – 0.67
Shih et al., 2018 [75]<br><br>United States of America	Sacramento Area Latino Study on Aging (SALSA); n=1,438 (58.4% F, mean age: 70 y).	Prospective longitudinal design (average follow-up: 6.5 y).	Age, sex, education, smoking status, hours of standing or walking at work, history of stroke, hypertension, cardiovascular disease, depressive symptoms, BMI, smoking, alcohol, nativity, area of residence, and type of occupation.	Self-reported physical activity, based on the metabolic equivalent of task (MET).	Incident dementia cases (n=136) based on standard clinical evaluation criteria.	Highest PA level:<br><br>HR, 0.7, 0.49 – 0.99
Larsson et al., 2018 [76]	Swedish Infrastructure for Medical Population-	Prospective longitudinal design	Age, sex, education, BMI, healthy diet, smoking, hypertension, alcohol and coffee consumption,	Self-reported physical activity; resulting from	Incident dementia cases (n=3,755) based on standard	Highest PA level:<br><br>HR, 0.99, 0.83 – 1.17

Sweden	based Life-course Environmental Research (SIMPLER); n=28,775 (46.6% F, mean age: 71.6 y).	(average follow-up: 12.6 y).	hypercholesterolemia, diabetes, and sleep duration.	walking/cycling 1.5+ hours per day, or leisure-time exercise 5+ hours per week).	clinical evaluation criteria.	
Shaaban et al., 2019 [77]<br><br>United States of America	Monongahela-Youghiogheny Healthy Aging Team (MYHAT); n=1,701 (62.3% F; mean age: 78 y).	Prospective longitudinal design (average follow-up: 3 y).	Unadjusted (did not include covariates).	Self-reported physical activity performed 3+ times per week.	Incident dementia cases (n=109) based on standard clinical evaluation criteria.	Highest PA level:<br><br>HR, 0.65, 0.47 – 0.9
Hansson et al., 2019 [78]<br><br>Sweden	Swedish Vasaloppet and participants in the Swedish population-based Malmo Diet and Cancer Study; n= 20,639 (60% W, mean age: 57.5 y).	Prospective longitudinal design (average follow-up: 15 y).	Age, sex, education, smoking, systolic blood pressure, BMI, alcohol, diabetes, cardiovascular disease, blood pressure-lowering medication, lipid-lowering medication, and physically heavy work.	Self-reported physical activity; resulting from the sum of the two assessments (5 years apart).	Incident dementia cases (n=1,375) based on standard clinical evaluation criteria.	Highest PA level:<br><br>HR, 0.9, 0.79 – 1.03
Najar et al., 2019 [79]<br><br>Sweden	Gothenburg study; n=800 W (average: 47.2 y).	Prospective longitudinal design (average follow-up: 31.5 y).	Age, cognitive activity, smoking, and socioeconomic status.	Self-reported physical activity, performed 3+ times per week.	Incident dementia cases (n=194) based on standard clinical evaluation criteria.	Highest PA level:<br><br>HR, 0.72, 0.50 – 1.04
Palta et al., 2019 [80]<br><br>United States of America	Atherosclerosis Risk in Communities (ARIC); n=10,705 (56% F, mean age: 60 y).	Prospective longitudinal design (average follow-up: 17.4 y).	Age, sex, education, race, APOE, smoking, household income, neighborhood socioeconomic status summary score at Visit 3, diabetes, hypertension, and BMI at Visit 4.	Self-reported physical activity at two time points; based on tertiles of metabolic equivalent of task (e.g., MET-min).	Incident dementia cases (n=1,063) based on standard clinical evaluation criteria.	Highest PA level:<br><br>HR, 0.71, 0.54 – 0.92
Zotcheva et al., 2019 [81]<br><br>Norway	Nord-Trondelag Health (HUNT) study; n= 28,916 (50.2% W, mean age: 43.4 y).	Prospective longitudinal design (average follow-up: 25.2 y).	Sex, education, marital status, smoking, alcohol, longstanding physical illness, and distress.	Self-reported physical activity, based on moderate-to-vigorous PA.	Incident dementia cases (n=359) based on standard clinical evaluation criteria.	Highest PA level:<br><br>HR, 0.81, 0.62 – 1.06
Kunutsor et al., 2020	Kuopio Ischaemic Heart Disease	Prospective longitudinal design	Age, BMI, systolic blood pressure, smoking, alcohol,	Self-reported physical activity;	Incident dementia cases (n=208)	Highest PA level:

[82]<br><br>Finland	(KIHD) study; n=2,394 M (mean age: 53 y).	(average follow-up: 24.9 y).	history of type 2 diabetes, total cholesterol, high-density lipoprotein cholesterol, history of coronary heart disease, and C-reactive protein.	based on the highest tertile resulting from the metabolic equivalent of task (MET) per day.	based on standard clinical evaluation criteria.	HR, 0.97, 0.69 – 1.38
Rolandi et al., 2020<br>[83]<br><br>Italy	Invecchiamento Cerebrale ad Abbiategrasso (InveCe.Ab) study; n=1,100 (54.1% F, mean age: 53.7 y).	Prospective longitudinal design (average follow-up: 6.9 y).	APOE, diabetes, heart disease, stroke, and delirium.	Self-reported physical activity, resulting from the inverse of physical inactivity.	Incident dementia cases (n=111) based on standard clinical evaluation criteria.	Highest PA level [i]:<br><br>HR, 0.75, 0.51 - 1.11
Dupre et al., 2020<br>[84]<br><br>France	Three-city cohort; n= 1,550 (63.6% F, mean age: 80 y).	Prospective longitudinal design (average follow-up: 4.6 y).	Age, center, sex, APOE, and education.	Self-reported physical activity performed 3+ times per week.	Incident dementia cases (n=117) based on standard clinical evaluation criteria.	Highest PA level:<br><br>HR, 1.33, 0.72 – 2.44
Wu et al., 2020<br>[85]<br><br>China	Shanghi Aging study; n=1,648 (54.5% F, mean age: 71.5 y).	Prospective longitudinal design (average follow-up: 5 y).	Age, sex, years of education, APOE, smoking, alcohol, hypertension, diabetes, and BMI.	Self-reported physical activity, based on the metabolic equivalent of task (MET).	Incident dementia cases (n=166) based on standard clinical evaluation criteria.	Highest PA level:<br><br>HR, 0.62, 0.44 – 0.89
Boongird et al., 2020<br>[86]<br><br>Thailand	Health Check Ubon Ratchathani Project; n=206,073 (53.6% F, mean age: 62.5 y).	Prospective longitudinal design (average follow-up: 6 y).	Age, hypertension, diabetes, BMI, waist circumference, obesity, central obesity, and alcohol.	Self-reported physical activity performed 5+ days per week.	Incident dementia cases (n=480) based on standard clinical evaluation criteria.	Highest PA level:<br><br>HR, 0.41, 0.26 – 0.66
Floud et al., 2020<br>[87]<br><br>United Kingdom	Million Women Study; n=1,136,846 W (mean age: 56 y).	Prospective longitudinal design (average follow-up: 18 y).	Year of birth, year reporting exposure, region of residence, educational qualifications, area deprivation, height, smoking, alcohol consumption, and use of menopausal hormones, and BMI.	Self-reported physical activity, performed 1+ times per week.	Incident dementia cases (n=18,695) based on standard clinical evaluation criteria.	Highest PA level [i]:<br><br>RR, 0.94, 1.02 – 1.12
Feter et al., 2021<br>[88]<br><br>United Kingdom	English Longitudinal Study of Ageing (ELSA); n=9,275 (56.2% F, mean age 63.8 y).	Prospective longitudinal design (average follow-up: 15 y).	Age, sex, ethnic group, education level, marital status, employment activity, and age x physical activity interaction, smoking, alcohol consumption, diabetes, emotional, nervous, or psychiatric problems, heart diseases (i.e., hypertension, angina, heart attack,	Self-reported physical activity performed 1+ times per week.	Incident dementia cases (n=631) based on standard clinical evaluation criteria.	Highest PA level:<br><br>HR, 0.22, 0.17 – 0.3

			heart murmur, abnormal heart rhythm, stroke), and cognitive function at baseline.			
Yoon et al., 2021 [89]<br><br>Korea	National Health Insurance Service (NHIS)–Senior database of Korea; n=62,286 (60.4% W, mean age: 73.2 y).	Prospective longitudinal design (average follow-up: 3.5 y).	Age, sex, BMI, Hospital Frailty Risk score, annual income, smoking, alcohol, hypertension, diabetes mellitus, dyslipidemia, chronic kidney disease, heart failure, vascular disease, prior ischemic stroke or transient ischemic attack, chronic obstructive pulmonary disease, and malignancy.	Self-reported physical activity, based on the metabolic equivalent of task (MET) per week.	Incident dementia cases (n=3,757) based on standard clinical evaluation criteria.	Highest PA level:<br><br>HR, 0.73, 0.65 – 0.83
Nabe-Nielsen et al., 2021 [90]<br><br>Denmark	Copenhagen Male Study (CMS); n=4,721 M (mean age: 49 y).	Prospective longitudinal design (average follow-up: 29.3 y).	Age, age at exposure assessment, marital status, socioeconomic position, calendar period, psychological stress, smoking, alcohol, BMI, and systolic and diastolic blood pressure.	Self-reported physical activity, performed 3+ times per week.	Incident dementia cases (n=697) based on standard clinical evaluation criteria.	Highest PA level:<br><br>RR, 0.8, 0.6 – 1.08
Stephan et al., 2021 [91]<br><br>United States of America	Health and Retirement Study (HRS); n=13,839 (60.0% F, mean age: 64.3y).	Prospective longitudinal design (maximum follow-up: 21.0 y).	Age, sex, race, educational level, ethnicity, self-reported diabetes, self-reported health, high blood pressure, depression, and smoking	Self-reported vigorous physical activity or exercised three times a week or more on average over the last 12 months.	Incident dementia cases (n=1,660) based on standard clinical evaluation criteria.	Highest PA level [i]:<br><br>HR, 0.98, 0.89 – 1.09
Yang et al., 2022 [92]<br><br>United States of America	The Southern Community Cohort Study; n=17,209 (63.1% W, mean age: 62.9 y).	Prospective longitudinal design (median follow-up: 4 y).	Enrollment age, time interval from enrollment to starting follow-up, calendar years of starting follow-up, sex, race, education, income, marital status, enrollment source, history of cardiometabolic disease (i.e., ischemic heart disease, diabetes, hypertension, and dyslipidemia), and history of depression.	Self-reported physical activity, ≥8.3 metabolic equivalent of task (MET) hours per week.	Incident Alzheimer's disease and related dementia cases (n=1,694) based on standard clinical evaluation criteria.	Highest PA level:<br><br>HR, 0.89, 0.77 – 1.03
Huang et al., 2022 [93]	The UK Biobank; n=431,924 (54.1% F, median age: 58.0 y).	Prospective longitudinal design (median follow-up: 9.04 y).	Age, sex, APOE ε4 status, and education.	Self-reported physical activity, ≥2400 metabolic equivalent (MET)-	Incident dementia cases (n=5,390) based on standard	Highest PA level:<br><br>HR, 0.85, 0.77 – 0.93

United Kingdom				min/week of total physical activity.	clinical evaluation criteria.	
i Risk estimates presented as their inverse to aid interpretation.<br>Abbreviations: CI, confidence interval; W, women; F, females; M, males or men; y, years; PA, physical activity; BMI, body mass index; APOE, apolipoprotein E ε4 allele; MET, metabolic equivalent of task; HR, Hazard Ratio; OR, Odds Ratio; RR, Risk Ratio.						
