# Supplementary material for: The Relationships between physical activity, sedentary behaviour, sleep, and dementia: A systematic review and meta-analysis of cohort studies
Source: PLoS One. 2026 Apr 8;21(4):e0343621. doi: 10.1371/journal.pone.0343621 (PMC13061222; doi:10.1371/journal.pone.0343621)
Supplement: S3 Table — Association between sleep duration and dementia risk. (PDF) [file pone.0343621.s003.pdf]

S3 Table. Study characteristics: sleep duration. Association between sleep duration and dementia risk.						
**Author and Country**	**Study and Sample**	**Design and Duration of Follow-up**	**Covariates**	**Sleep Measure**	**Incident Dementia**	**Risk Estimate (95%CI)**
Benito-Leon et al., 2009 [94]<br><br>Spain	Neurological Disorders of Central Spain (NEDICES); n=3,286 (56.9% F; mean age: 73.2 y).	Prospective longitudinal design (average follow-up: 3.2 y).	Age, education, smoking, and alcohol.	Self-reported nightly sleep duration (hours) was categorized (≤ 5, 6, 7, 8, ≥ 9).	Incident dementia cases (n=140) based on standard clinical evaluation criteria.	Short Sleep:<br>RR, 1.87, 0.85 – 4.15<br><br>Long Sleep:<br>RR, 2.18, 1.09 – 4.37
Virta et al., 2013 [95]<br><br>Finland	Finnish Twin study; n=2,336 (47.9% W; mean age: 73.6 y).	Prospective longitudinal design (average follow-up: 22.5 y).	Age, sex, education, APOE status, follow-up, life satisfaction, obesity, hypertension, physical inactivity, and alcohol.	Self-reported nightly sleep duration (hours) was categorized (< 7, 7 to < 8, ≥ 8).	Incident dementia cases (n=170) based on standard clinical evaluation criteria.	Short Sleep:<br>OR, 1.5, 0.92 – 2.45<br><br>Long Sleep:<br>OR, 1.75, 1.07 – 2.88
Chen et al., 2016 [96]<br><br>United States of America	Women's Health Initiative Memory Study (WHIMS); n=7,444 W (mean age: 70.1 y).	Prospective longitudinal design (average follow-up: 7.3 to 7.7 y).	Age, race, socioeconomic status, smoking, alcohol, physical activity, depression, previous hormone therapy use, BMI, prior cardiovascular disease history, hypertension, diabetes mellitus, and hypercholesterolemia.	Self-reported nightly sleep duration (hours) was categorized (≤ 6, 7, ≥ 8).	Incident dementia cases (n=549) based on standard clinical evaluation criteria.	Short Sleep:<br>HR, 1.36, 1.09 – 1.71<br><br>Long Sleep:<br>HR, 1.27, 0.98 – 1.64
Diem et al., 2016 [97]<br><br>United States of America	Study of Osteoporotic Fractures (SOF); n=1,245 W (mean age: 82.6 y).	Prospective longitudinal design (average follow-up: 4.9 y).	Age, race, clinic, and education.	Self-reported nightly sleep duration (hours) was categorized (< 6, 6 to <7.5, ≥ 7.5).	Incident dementia cases (n=473) based on standard clinical evaluation criteria.	Short Sleep:<br>OR, 0.85, 0.63 – 1.16<br><br>Long Sleep:<br>OR, 0.93, 0.69 – 1.25
Bokenberger et al., 2016 [98]<br><br>Sweden	Swedish Screening Across the Lifespan Twin (SALT); n=11,247 (56.4% W, mean age: 72.5 y).	Prospective longitudinal design (average follow-up: 14.3 y).	Follow-up time, sex, and education with age as the underlying timescale.	Self-reported nightly sleep duration (hours) was categorized (≤ 6, ≥ 9).	Incident dementia cases (n=1,850) based on standard clinical evaluation criteria.	Short Sleep:<br>HR, 1.74, 1.19 – 2.55<br><br>Long Sleep:<br>HR, 1.18, 1.01 – 1.38
Sabia et al., 2017 [99]<br><br>United Kingdom	Whitehall II study; n=7,959, (32.8% F, mean age: 50.6 y).	Prospective longitudinal design (average follow-up: 25 y).	Age, sex, ethnicity, education, marital status, alcohol, physical activity, smoking, fruit and vegetable consumption, BMI, hypertension, diabetes, cardiovascular disease,	Self-reported nightly sleep duration (hours) was categorized (≤ 6, 7, ≥ 8).	Incident dementia cases (n=521) based on standard clinical evaluation criteria.	Short Sleep:<br>HR, 1.22, 1.01 – 1.48<br><br>Long Sleep:<br>HR, 1.25, 0.98 – 1.60

			depression, and central nervous system medications.			
Luojus et al., 2017 [100]<br><br>Finland	Kuopio Ischemic Heart Disease (KIHD) study; n=2,386 M (mean age: 53 y).	Prospective longitudinal design (average follow-up: 21.9 y).	Age, examination year, depression, physical activity, alcohol, smoking, systolic blood pressure, BMI, low-density lipoprotein and high-density lipoprotein cholesterol, high-sensitivity C-reactive protein, cardiovascular disease history, education, and living alone.	Self-reported nightly sleep duration (hours) was categorized (≤ 6.5, 7 to <8, ≥ 8.5).	Incident dementia cases (n=287) based on standard clinical evaluation criteria.	Short Sleep:<br>RR, 1.15, 0.85 – 1.56<br><br>Long Sleep:<br>RR, 0.83, 0.55 – 1.23
Westwood et al., 2017 [101]<br><br>United States of America	Framingham Heart Study; n=2,457 (57% F; mean age: 72 y).	Prospective longitudinal design (average follow-up: 10 y).	Age, sex, education, APOE status, and homocysteine	Self-reported nightly sleep duration (hours) was categorized (< 6, 6 to 9, > 9).	Incident dementia cases (n=234) based on standard clinical evaluation criteria.	Short Sleep:<br>HR, 0.9, 0.58 – 1.38<br><br>Long Sleep:<br>HR, 2.01, 1.24 – 3.26
Lutsey et al., 2018 [102]<br><br>United States of America	Atherosclerosis Risk in Communities (ARIC); n=1,667 (53% F; mean age: 61.4 y).	Prospective longitudinal design (average follow-up: 15 y).	Age, sex, center, education, and APOE, BMI, smoking, and leisure time physical activity, diabetes, antihypertensive medications, C-reactive protein, and systolic blood pressure.	Self-reported nightly sleep duration (hours) was categorized (< 7, 7 to < 8, 8 to 9, ≥ 9).	Incident MCI/dementia cases (n=269) based on standard clinical evaluation criteria.	Short Sleep:<br>RR, 1.89, 1.01 – 3.51<br><br>Long Sleep:<br>RR, 1.53, 0.69 – 3.39
Sindi et al., 2018 [103]<br><br>Finland and Sweden	Kungsholmen Project (KP), H70 study, Cardiovascular Risk Factors study, Aging and Dementia (CAIDE); n=1,446; late-life (60.4% W, mean ages: 70 y for CAIDE/H70).	Prospective longitudinal design (average follow-up: 5 to 9 y).	Age, sex, education, follow-up time, study, alcohol consumption, smoking, physical activity, cohabitant status, APOE status, cardio/cerebrovascular conditions and hypnotics, hopelessness.	Self-reported nightly sleep duration (hours) was categorized (≤ 6, ≥ 9).	Incident dementia cases (H70 study, n=19, out of 437) based on standard clinical evaluation criteria.	Short Sleep:<br>OR, 0.74, 0.36 – 1.53<br><br>Long Sleep:<br>OR, 3.98, 1.87 – 8.48
Larsson et al., 2018 [104]<br><br>Sweden	Swedish Infrastructure for Medical Population-Based Life-Course and Environmental Research (SIMPLER);	Prospective longitudinal design (average follow-up: 12.6 y).	Age, sex, education, BMI, healthy diet, smoking, hypertension, alcohol and coffee consumption, hypercholesterolemia, diabetes, and physical activity.	Self-reported nightly sleep duration (hours) was categorized (≤ 6, 6 to 7, 7 to 9, > 9).	Incident dementia cases (n=3,755) based on standard clinical evaluation criteria.	Short Sleep:<br>HR, 1.05, 0.93 – 1.19<br><br>Long Sleep:<br>HR, 1.44, 1.11 – 1.86

	n=28,775 (46.6% F, mean age: 71.6 y)					
Ohara et al., 2018 [105]<br><br>Japan	Hisayama study; n=1,517 (56% F, mean age: 70 y).	Prospective longitudinal design (average follow-up: 8.8 y).	Age, sex, education, systolic blood pressure, antihypertensive agent, diabetes mellitus, use of hypnotics, hypercholesterolemia, BMI, electrocardiographic abnormalities, history of stroke, smoking habits, alcohol intake, and regular exercise.	Self-reported nightly sleep duration (hours) was categorized (< 5, 5 to 6.9, 7 to 7.9, 8 to 9.9, ≥ 10).	Incident dementia cases (n=294) based on standard clinical evaluation criteria.	Short Sleep:<br>HR, 2.64, 1.38 – 0.05<br><br>Long Sleep:<br>HR, 1.57, 1.15 – 2.16
Lu et al., 2018 [106]<br><br>Japan	Ohsaki Cohort study; n= 7,422 (55.9% F, mean age: 74.6 y).	Prospective longitudinal design (average follow-up: 5.7 y).	Age, sex, sleep duration in 1994, BMI, history of diseases (stroke, hypertension, myocardial infarction, diabetes, or hyperlipidemia), smoking, alcohol, education, pain, psychological distress score, and time spent walking.	Self-reported nightly sleep duration (hours) was categorized (≤ 6, 7 to < 8, ≥ 9).	Incident dementia cases (n=688) based on standard clinical evaluation criteria.	Short Sleep:<br>HR, 0.98, 0.61 – 1.57<br><br>Long Sleep:<br>HR, 1.01, 0.75 – 1.34
Uwaka et al., 2022 [107]<br><br>Japan	New Integrated Suburban Seniority Investigation (NISSIN) project, Japan; n=1,954 (48.5% F; mean age: 64 y).	Prospective longitudinal design (average follow-up: 15.6 y).	Year of participation, sex, marital status, working status, education, smoking, alcohol, BMI, daily walking, depressive tendencies, and functional capacity.	Self-reported nightly sleep duration (hours) was categorized (≤ 6, 6 to 7.9, ≥ 8).	Incident dementia cases (n=260) based on standard clinical evaluation criteria.	Short Sleep:<br>HR, 1.7, 1.02 – 2.83<br><br>Long Sleep:<br>HR, 1.34, 0.84 – 1.95
Huang et al., 2022 [93]<br><br>United Kingdom	The UK Biobank; n=431,924 (54.1% F, median age: 58.0 y).	Prospective longitudinal design (median follow-up: 9.04 y).	Age, sex, APOE ε4 status, and education.	Self-reported nightly sleep duration (hours) was categorized (≤ 6, 7, ≥ 8).	Incident dementia cases (n=5,390) based on standard clinical evaluation criteria.	Short Sleep:<br>HR, 1.19, 1.07 – 1.31<br><br>Long Sleep:<br>HR, 1.23, 1.13 – 1.34
Liu et al., 2022 [108]<br><br>China	Communities in western Shandong cohort study; n=1,982 (% F data not available, age: 70.1 y)	Prospective longitudinal design (average follow-up: 3.7 y).	Age, sex, education, BMI, alcohol consumption, smoking, hypertension, diabetes, dyslipidemia, coronary heart disease, stroke, and APOE genotype.	Self-reported nightly sleep duration (hours) was categorized (< 7, 7–8, > 8).	Incident dementia cases (n=97) based on standard clinical evaluation criteria.	Short Sleep $^{\alpha}$:<br>HR, 1.25, 0.78 – 2.0<br><br>Long Sleep $^{\alpha}$:<br>HR, 1.76, 1.05 – 2.95

Wong et al., 2023 [109]<br><br>United Kingdom	Million Women Study; n=830,716 W (mean age: 60 y).	Prospective longitudinal design (average follow-up: 17 y).	Deprivation, education, exercise, BMI, smoking status, alcohol, menopausal hormones, paid work, marital status, depression/anxiety, diabetes, high blood pressure, and self-rated health.	Self-reported nightly sleep duration (hours) was categorized (< 7, 7 to <8, ≥ 8).	Incident dementia cases (n=34,576) based on standard clinical evaluation criteria.	Short Sleep:<br>RR, 1.12, 1.06 – 1.19<br><br>Long Sleep:<br>RR, 1.08, 1.0 – 1.16

α Risk estimate presented as Subdistribution Hazard Ratio.
Abbreviations: CI, confidence interval; W, women; F, females; M, males or men; y, years; BMI, body mass index; APOE, apolipoprotein E ε4 allele; HR, Hazard Ratio; OR, Odds Ratio; RR, Risk Ratio; MCI, mild cognitive impairment.
