# Supplementary material for: The Relationships between physical activity, sedentary behaviour, sleep, and dementia: A systematic review and meta-analysis of cohort studies
Source: PLoS One. 2026 Apr 8;21(4):e0343621. doi: 10.1371/journal.pone.0343621 (PMC13061222; doi:10.1371/journal.pone.0343621)
Supplement: S4 Table — Association between sedentary behaviour and dementia risk. (PDF) [file pone.0343621.s004.pdf]

S4 Table. Study characteristics: sedentary behaviour. Association between sedentary behaviour and dementia risk.						
**Author and Country**	**Study and Sample**	**Design and Duration of Follow-up**	**Covariates**	**Sedentary Behaviour Measure**	**Incident Dementia**	**Risk Estimate (95%CI)**
Tan et al., 2017 [68]<br><br>United States of America	Framingham Study Original and Offspring; n=3,714 (54.4% F; mean age: 70.5 y).	Prospective longitudinal design (average follow-up: 7.5 y).	Age, sex, high school degree, APOE *e4 allele status*, log plasma homocysteine, systolic blood pressure, diastolic blood pressure, antihypertensive medication, total cholesterol, current smoking, prevalent cardiovascular disease, diabetes, stroke, and atrial fibrillation.	Self-reported sedentary hours per day, categorized into quintiles.	Incident dementia cases (n=236) based on standard clinical evaluation criteria.	Highest Sedentary level:<br><br>HR, 1.18, 0.84 – 1.67
Huang et al., 2023 [110]<br><br>United Kingdom	The UK Biobank; n=201,624 (47.6% F, mean age: 56.1 y).	Prospective longitudinal design (median follow-up: 11.8 y).	Age, sex, household income, loneliness, Townsend index, family history of dementia, current drinker, BMI level, hearing problem, hypertension, diabetes, loneliness, and sleep duration.	Self-reported sedentary hours daily (included watching TV, playing video game, and driving a car, calculated as the sum of these activities), categorized into quintiles.	Incident dementia cases (n=1,841) based on standard clinical evaluation criteria.	Highest Sedentary level:<br><br>HR, 1.23, 1.08 – 1.41
Du et al., 2024 [111]<br><br>Japan	Japan Gerontological Evaluation Study (JAGES); n=90,471 (54.0% F; median age: 73 y).	Prospective longitudinal design (median follow-up: 3.4 y).	Sex, age, marital status, educational attainment, family equivalent income, employment, BMI, comorbidity, self-rated health, alcohol, smoking, frequency of eating meat or fish, frequency of eating fruits and vegetables, and social isolation.	Self-reported sedentary hours per day, categorized as <3, 3 to <8, and ≥8 hours per day.	Incident dementia cases (n=4,135) based on standard clinical evaluation criteria.	Highest Sedentary level:<br><br>HR, 1.33, 1.17 – 1.51
Abbreviations: CI, confidence interval; W, women; F, females; M, males or men; y, years; BMI, body mass index; APOE, apolipoprotein E ε4 allele; HR, Hazard Ratio; OR, Odds Ratio; RR, Risk Ratio.						
