# Supplementary material for: The Relationships between physical activity, sedentary behaviour, sleep, and dementia: A systematic review and meta-analysis of cohort studies
Source: PLoS One. 2026 Apr 8;21(4):e0343621. doi: 10.1371/journal.pone.0343621 (PMC13061222; doi:10.1371/journal.pone.0343621)
Supplement: S5 Fig — Graphical representation of publication bias for associations between physical activity and incident dementia. (PDF) [file pone.0343621.s005.pdf]

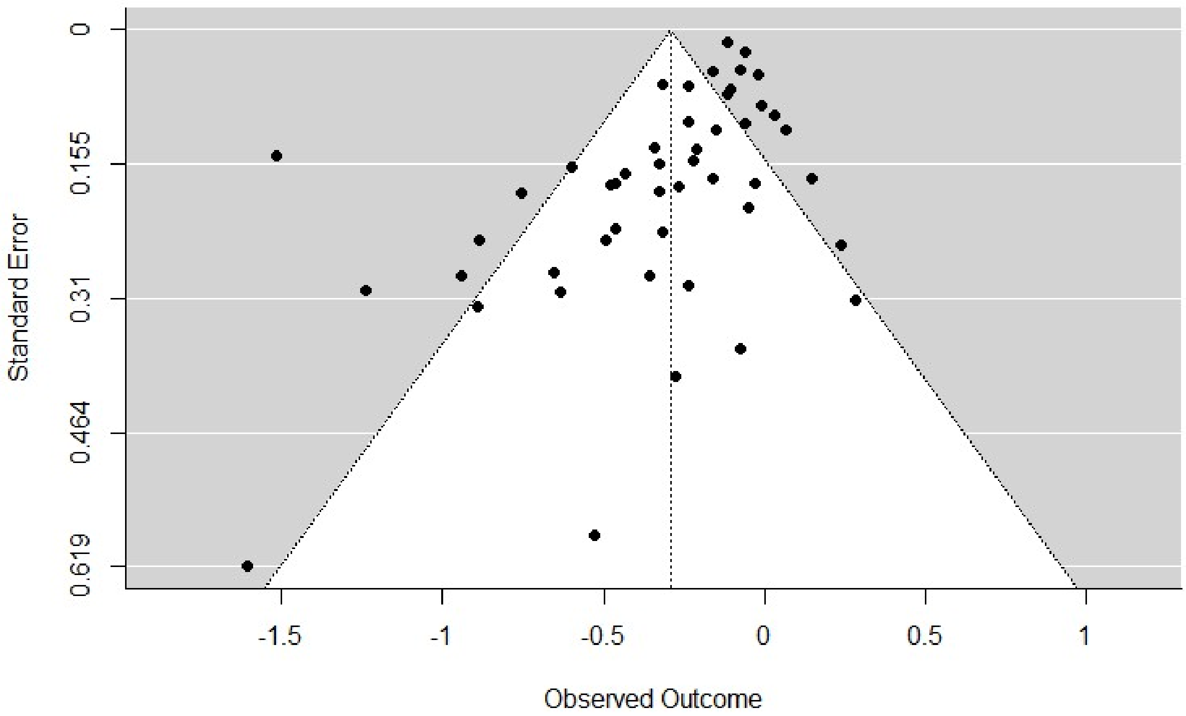

S5 Fig. Funnel plot: physical activity. Graphical representation of publication bias for associations between physical activity and incident dementia.

Note: An uneven or asymmetrical cluster of dots with outliers far from the expected range suggests a higher chance of publication bias where studies with certain results (often positive) are more likely to be published..
