# Supplementary material for: The Relationships between physical activity, sedentary behaviour, sleep, and dementia: A systematic review and meta-analysis of cohort studies
Source: PLoS One. 2026 Apr 8;21(4):e0343621. doi: 10.1371/journal.pone.0343621 (PMC13061222; doi:10.1371/journal.pone.0343621)
Supplement: S6 Fig — Graphical representation of publication bias for associations between short sleep and incident dementia. (PDF) [file pone.0343621.s006.pdf]

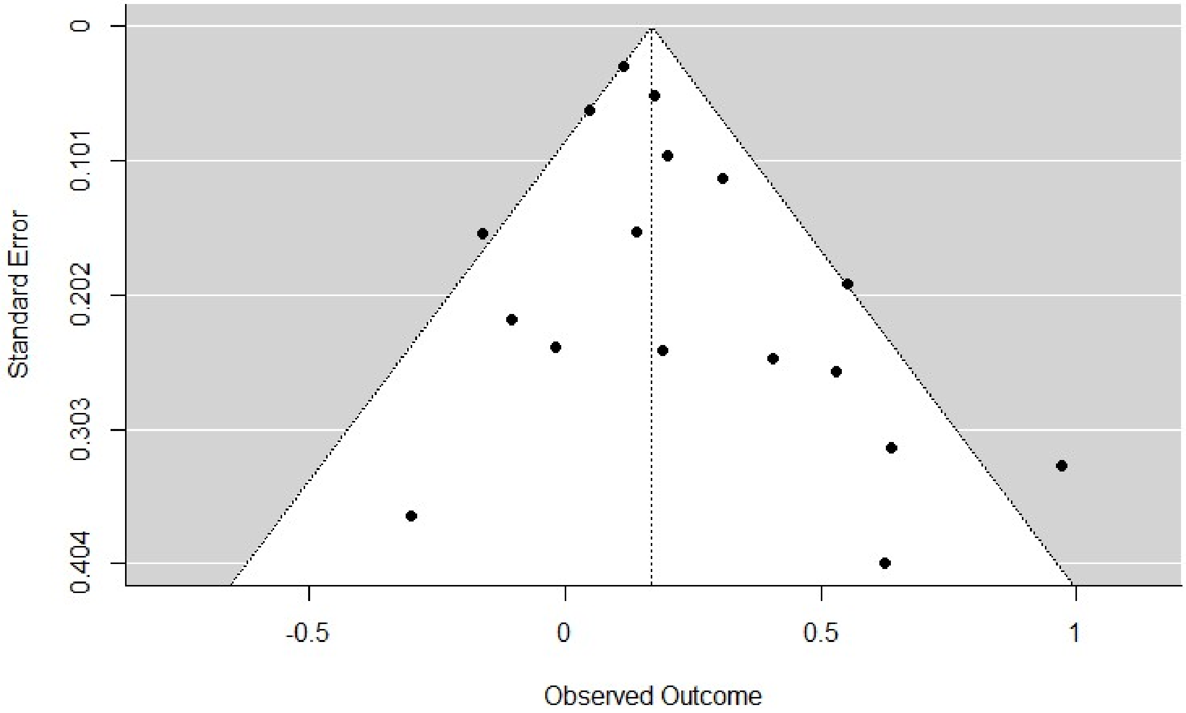

S6 Fig. Funnel plot: short sleep. Graphical representation of publication bias for associations between short sleep and incident dementia.

Note: An even or symmetrical distribution of dots inside the funnel indicates consistency in the findings and publication bias is less likely to influence the results.
