# Supplementary material for: The Relationships between physical activity, sedentary behaviour, sleep, and dementia: A systematic review and meta-analysis of cohort studies
Source: PLoS One. 2026 Apr 8;21(4):e0343621. doi: 10.1371/journal.pone.0343621 (PMC13061222; doi:10.1371/journal.pone.0343621)
Supplement: S8 Fig — Graphical representation of publication bias for associations between sedentary behaviour and incident dementia. (PDF) [file pone.0343621.s008.pdf]

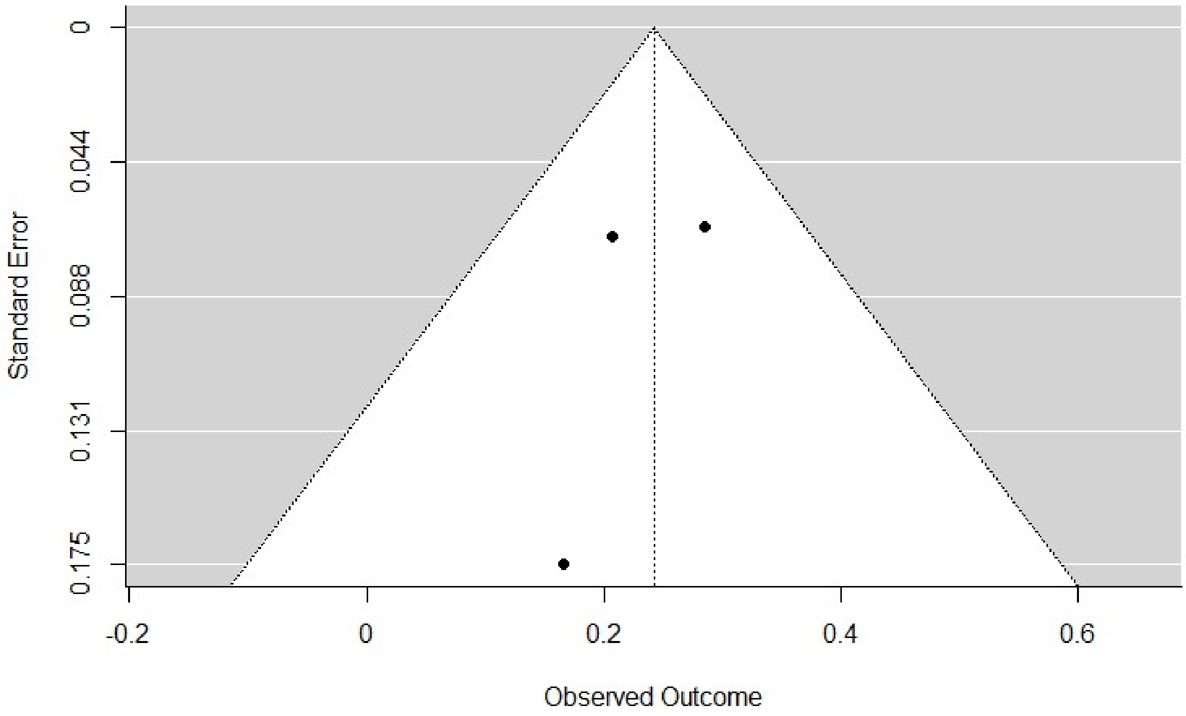

S8 Fig. Funnel plot: sedentary behaviour. Graphical representation of publication bias for associations between sedentary behaviour and incident dementia.

Note: With only three studies, the funnel plot provides limited insight, and potential publication bias should be interpreted with caution.
