# Supplementary material for: The Relationships between physical activity, sedentary behaviour, sleep, and dementia: A systematic review and meta-analysis of cohort studies
Source: PLoS One. 2026 Apr 8;21(4):e0343621. doi: 10.1371/journal.pone.0343621 (PMC13061222; doi:10.1371/journal.pone.0343621)
Supplement: S9 Table — Association between physical activity and dementia. (PDF) [file pone.0343621.s009.pdf]

S9 Table. Risk of bias assessment: physical activity. Association between physical activity and dementia.

Author	Year	Confounding[1]	Selection[2]	Classification[3]	Deviations[4]	Missing[5]	Measurement[6]	Reporting[7]	Overall[8]
Yoshitake	1995	Moderate	Low	Low	Low	Low	Low	Low	Moderate
Laurin	2001	Low	Low	Low	Low	Moderate	Low	Low	Moderate
Verghese	2003	Moderate	Low	Low	Low	Low	Low	Low	Moderate
Abbot	2004	Moderate	Low	Low	Low	Moderate	Low	Low	Moderate
Podwils	2005	Low	Low	Low	Low	Moderate	Low	Low	Moderate
Ravaglia	2008	Low	Low	Low	Low	Low	Low	Low	Low
Scarmeas	2009	Low	Low	Low	Low	Moderate	Low	Low	Moderate
Chang	2010	Moderate	Low	Low	Low	Moderate	Low	Low	Moderate
Sattler	2011	Serious	Low	Low	Low	Moderate	Low	Low	Serious
Bowen	2012	Moderate	Low	Low	Low	Low	Low	Low	Moderate
Buchman	2012	Low	Low	Low	Low	Moderate	Low	Low	Moderate
Verdelho	2012	Serious	Low	Low	Low	NI	Low	Low	Serious
Elwood	2013	Serious	Low	Low	Low	Low	Low	Low	Serious
Gray	2013	Moderate	Low	Low	Low	Moderate	Low	Low	Moderate
De Bruijn	2013	Moderate	Low	Low	Low	Moderate	Low	Low	Moderate
Tolppanen	2014	Low	Low	Low	Low	Moderate	Low	Low	Moderate
Wang	2014	Moderate	Low	Low	Low	Moderate	Low	Low	Moderate
Luck	2014	Low	Low	Low	Low	Moderate	Low	Low	Moderate
Llamas-Velasco	2015	Moderate	Low	Low	Low	NI	Low	Low	Moderate
Paganini-Hill	2016	Moderate	Low	Low	Low	Moderate	Low	Low	Moderate
Hessler	2016	Moderate	Low	Low	Low	Moderate	Low	Low	Moderate
Neergaard	2016	Low	Low	Moderate	Low	Moderate	Low	Low	Moderate
Tan	2017	Moderate	Low	Low	Low	Moderate	Low	Low	Moderate
Muller	2017	Moderate	Moderate	Low	Low	Low	Low	Low	Moderate
Gross	2017	Serious	Low	Low	Low	Moderate	Low	Low	Serious
Sabia	2017	Serious	Low	Low	Low	NI	Low	Low	Serious
Tomata	2017	Moderate	Low	Low	Low	Moderate	Low	Low	Moderate
Deckers	2017	Serious	Low	Low	Low	Low	Low	Low	Serious
Hwangbo	2017	Serious	Low	Moderate	Low	Moderate	Low	Low	Serious
Shih	2018	Low	Low	Low	Low	Low	Low	Low	Low
Shakersain	2018	Low	Low	Low	Low	Low	Low	Low	Low
Larsson	2018	Serious	Low	Low	Low	Low	Low	Low	Serious
Shaaban	2019	Moderate	Low	Low	Low	Low	Low	Low	Moderate
Hansson	2019	Moderate	Moderate	Low	Low	Moderate	Low	Low	Moderate
Najar	2019	Low	Low	Low	Low	Moderate	Low	Low	Moderate

Palta	2019	Serious	Low	Low	Low	Moderate	Low	Low	Serious
Zotcheva	2019	Serious	Low	Low	Low	Moderate	Low	Low	Serious
Kunutsor	2020	Moderate	Low	Low	Low	Low	Low	Low	Moderate
Rolandi	2020	Low	Low	Low	Low	Moderate	Low	Low	Moderate
Dupre	2020	Low	Low	Low	Low	Moderate	Low	Low	Moderate
Wu	2020	Low	Low	Low	Low	Moderate	Low	Low	Moderate
Boongird	2020	Moderate	Low	Low	Low	Moderate	Low	Low	Moderate
Floud	2020	Serious	Low	Low	Low	Low	Low	Low	Serious
Feter	2021	Moderate	Low	Low	Low	Moderate	Low	Low	Moderate
Yoon	2021	Moderate	Low	Low	Low	Low	Low	Low	Moderate
Nabe-Nielsen	2021	Moderate	Low	Low	Low	Moderate	Low	Low	Moderate
Stephan	2021	Low	Low	Low	Low	Moderate	Low	Low	Moderate
Yang	2022	Moderate	Moderate	Low	Low	Low	Low	Low	Moderate
Huang	2022	Serious	Moderate	Low	Low	Low	Low	Low	Serious

Abbreviation: NI, no information.
Risk of Bias Study Domains:

1) Bias due to confounding
2) Bias in selection of participants into the study
3) Bias in classification of interventions
4) Bias due to deviations from intended interventions
5) Bias due to missing data
6) Bias in measurement of outcomes
7) Bias in selection of the reported result
8) Overall Bias
