# Supplementary material for: The Relationships between physical activity, sedentary behaviour, sleep, and dementia: A systematic review and meta-analysis of cohort studies
Source: PLoS One. 2026 Apr 8;21(4):e0343621. doi: 10.1371/journal.pone.0343621 (PMC13061222; doi:10.1371/journal.pone.0343621)
Supplement: S10 Table — Association between sleep duration and dementia. (PDF) [file pone.0343621.s010.pdf]

S10 Table. Risk of bias assessment: sleep duration. Association between sleep duration and dementia.

Author	Year	Confounding[1]	Selection[2]	Classification[3]	Deviations[4]	Missing[5]	Measurement[6]	Reporting[7]	Overall[8]
Benito-Leon	2009	Serious	Low	Low	Low	Moderate	Low	Low	Serious
Virta	2013	Low	Low	Low	Low	Moderate	Low	Low	Moderate
Chen	2016	Low	Low	Low	Low	Low	Low	Low	Low
Bokenberger	2016	Moderate	Low	Low	Low	Moderate	Low	Low	Moderate
Diem	2016	Moderate	Low	Low	Low	Moderate	Low	Low	Moderate
Sabia	2017	Moderate	Low	Low	Low	Moderate	Low	Low	Moderate
Luojus	2017	Low	Low	Low	Low	Moderate	Low	Low	Moderate
Westwood	2017	Low	Low	Low	Low	Moderate	Low	Low	Moderate
Larsson	2018	Serious	Low	Low	Low	Low	Low	Low	Serious
Lutsey	2018	Moderate	Low	Low	NI	Serious	Low	Low	Serious
Sindi	2018	Moderate	NI	Low	Low	Moderate	Low	Low	Moderate
Ohara	2018	Moderate	Low	Low	Low	Low	Low	Low	Moderate
Lu	2018	Moderate	Low	Low	Low	Moderate	Low	Low	Moderate
Uwaka	2022	Moderate	Low	Low	Low	Low	Low	Low	Moderate
Huang	2022	Serious	Moderate	Low	Low	Low	Low	Low	Serious
Liu	2022	Moderate	Low	Low	Low	Moderate	Low	Low	Moderate
Wong	2023	Moderate	Low	Low	Low	Moderate	Low	Low	Moderate

Abbreviation: NI, no information.

Risk of Bias Study Domains:

1) Bias due to confounding
2) Bias in selection of participants into the study
3) Bias in classification of interventions
4) Bias due to deviations from intended interventions
5) Bias due to missing data
6) Bias in measurement of outcomes
7) Bias in selection of the reported result
8) Overall Bias
