# Supplementary material for: The Relationships between physical activity, sedentary behaviour, sleep, and dementia: A systematic review and meta-analysis of cohort studies
Source: PLoS One. 2026 Apr 8;21(4):e0343621. doi: 10.1371/journal.pone.0343621 (PMC13061222; doi:10.1371/journal.pone.0343621)
Supplement: S11 Table — Association between sedentary time and dementia. (PDF) [file pone.0343621.s011.pdf]

S11 Table. Risk of bias assessment: sedentary time. Association between sedentary time and dementia.									
**Author**	**Year**	**Confounding[1]**	**Selection[2]**	**Classification[3]**	**Deviations[4]**	**Missing[5]**	**Measurement[6]**	**Reporting[7]**	**Overall[8]**
Tan	2017	Moderate	Low	Low	Low	Moderate	Low	Low	Moderate
Huang	2023	Serious	Low	Low	Low	Moderate	Low	Low	Serious
Du	2024	Serious	Low	Low	Low	Low	Low	Low	Serious

Abbreviation: NI, no information.
Risk of Bias Study Domains:

1) Bias due to confounding
2) Bias in selection of participants into the study
3) Bias in classification of interventions
4) Bias due to deviations from intended interventions
5) Bias due to missing data
6) Bias in measurement of outcomes
7) Bias in selection of the reported result
8) Overall Bias
